# Long-term microdystrophin gene therapy is effective in a canine model of Duchenne muscular dystrophy

**DOI:** 10.1038/ncomms16105

**Published:** 2017-07-25

**Authors:** Caroline Le Guiner, Laurent Servais, Marie Montus, Thibaut Larcher, Bodvaël Fraysse, Sophie Moullec, Marine Allais, Virginie François, Maeva Dutilleul, Alberto Malerba, Taeyoung Koo, Jean-Laurent Thibaut, Béatrice Matot, Marie Devaux, Johanne Le Duff, Jack-Yves Deschamps, Inès Barthelemy, Stéphane Blot, Isabelle Testault, Karim Wahbi, Stéphane Ederhy, Samia Martin, Philippe Veron, Christophe Georger, Takis Athanasopoulos, Carole Masurier, Federico Mingozzi, Pierre Carlier, Bernard Gjata, Jean-Yves Hogrel, Oumeya Adjali, Fulvio Mavilio, Thomas Voit, Philippe Moullier, George Dickson

**Affiliations:** 1 Atlantic Gene Therapies, INSERM UMR 1089, Université de Nantes, CHU de Nantes, IRS2 Nantes Biotech, 22, bd Bénoni Goullin, 44200 Nantes, France; 2 Généthon, 1 bis rue de l’Internationale, 91000 Evry, France; 3 Institute I-Motion, Hôpital Armand Trousseau, 26 avenue du Dr A. Netter, 75571 Paris, France; 4 Atlantic Gene Therapies, INRA UMR 703, ONIRIS, La Chantrerie, BP 40706, 44307 Nantes, France; 5 Atlantic Gene Therapies, Centre de Boisbonne, ONIRIS, La Chantrerie, BP 40706, 44307 Nantes, France; 6 School of Biological Sciences, Royal Holloway, University of London, Egham, Surrey TW20 0EX, UK; 7 Institut de Myologie, Laboratoire RMN, AIM & CEA, 47 bd de l’Hôpital, 75013 Paris, France; 8 Université Paris-Est, Ecole Nationale Vétérinaire d'Alfort, 7 avenue du Général de Gaulle, 94704 Maisons-Alfort, France; 9 INSERM U955-E10 Biology of the NeuroMuscular System, Faculté de médecine, 8 rue du Général Sarrail, 94000 Créteil, France; 10 Centre Hospitalier Vétérinaire Atlantia, 22 rue René Viviani, 44200 Nantes, France; 11 Service de cardiologie, AP-HP, Cochin Hospital–Université Paris Descartes-Sorbonne Paris Cité–Institut de Myologie, Reference Center for Muscle Diseases, 27 rue du Faubourg St Jacques, 75014 Paris, France; 12 Service de cardiologie, hôpital Saint-Antoine, AP-HP, 184 rue du Faubourg St Antoine, 75012 Paris, France; 13 Faculty of Science and Engineering, University of Wolverhampton, Wulfruna Street, Wolverhampton WV1 1LY, UK; 14 Institut de Myologie, Neuromuscular Physiology and Evaluation Laboratory, 47 bd de l’Hôpital, 75013 Paris, France; 15 NIHR Biomedical Research Centre, UCL Institute of Child Health/Great Ormond Street Hospital NHS Trust, London WC1N 3JH, UK; 16 Department of Molecular Genetics and Microbiology, University of Florida, Gainesville, Florida 32611, USA

## Abstract

Duchenne muscular dystrophy (DMD) is an incurable X-linked muscle-wasting disease caused by mutations in the dystrophin gene. Gene therapy using highly functional microdystrophin genes and recombinant adeno-associated virus (rAAV) vectors is an attractive strategy to treat DMD. Here we show that locoregional and systemic delivery of a rAAV2/8 vector expressing a canine microdystrophin (cMD1) is effective in restoring dystrophin expression and stabilizing clinical symptoms in studies performed on a total of 12 treated golden retriever muscular dystrophy (GRMD) dogs. Locoregional delivery induces high levels of microdystrophin expression in limb musculature and significant amelioration of histological and functional parameters. Systemic intravenous administration without immunosuppression results in significant and sustained levels of microdystrophin in skeletal muscles and reduces dystrophic symptoms for over 2 years. No toxicity or adverse immune consequences of vector administration are observed. These studies indicate safety and efficacy of systemic rAAV-cMD1 delivery in a large animal model of DMD, and pave the way towards clinical trials of rAAV–microdystrophin gene therapy in DMD patients.

## Abstract (web summary)

Duchenne muscular dystrophy is a progressive degenerative disease of muscles caused by mutations in the dystrophin gene. Here the authors use AAV vectors to deliver microdystrophin to dogs with muscular dystrophy, and show restoration of dystrophin expression and reduction of symptoms up to 26 months of age.

Duchenne muscular dystrophy (DMD) is an X-linked inherited disease affecting ∼1:5,000 male births, leading to a highly debilitating and ultimately life-limiting muscle-wasting condition. DMD is caused by mutations in the gene coding for dystrophin, a cytoskeletal protein that is critical to the stability and function of myofibres in skeletal and cardiac muscle^1^,^2^. Dystrophin establishes a mechanical link between cytoskeletal actin and the extracellular matrix in muscle fibres through the dystrophin-associated protein complex, and when dystrophin is absent the mechanical and signalling functions of the costamer are compromised^3^. DMD-affected boys develop muscle weakness during the first years of life, and although palliative treatments are available (essentially physiotherapy, assisted ventilation and glucocorticoids) they become wheelchair-bound generally before the age of 15 years. Serious, life-threatening muscle wasting and respiratory and cardiac complications arise in late teens, and patients rarely survive into their fourth decade^4^,^5^.

Gene transfer therapy to restore dystrophin expression is considered a promising approach for the treatment of DMD. Recombinant adeno-associated virus (rAAV) vectors are particularly efficient in transducing skeletal muscle fibres and cardiomyocytes when packaged with the appropriate capsid^6^,^7^,^8^,^9^,^10^,^11^, and allow long-term *in vivo* transgene expression^12^,^13^. However, the full-length dystrophin complementary DNA (cDNA) is ∼14 kb in length and greatly exceeds the packaging capacity of a single rAAV vector. Shortened transgenes, coding for partially functional microdystrophins (MDs) that contain essential domains of the dystrophin protein, have however been generated to be compatible with rAAV vectors^14^,^15^. The principle of using MDs as therapeutic transgenes arose from the concept that Becker muscular dystrophy patients exhibiting natural in-frame deletions/mutations in their *DMD* gene exhibit a milder dystrophinopathy^14^,^16^. Several studies have shown body-wide expression and therapeutic efficacy of MDs in *mdx* mice following a single systemic administration of rAAV-MD vectors^17^,^18^,^19^. In particular, a MD variant, termed MD1, was optimized for mRNA stability and translation efficiency^20^, and packaged in rAAV vectors under the control of the synthetic, muscle- and cardiac-restricted promoter Spc5.12 (ref. 21). Intramuscular and systemic administration of rAAV-Spc5.12-MD1 vectors was previously shown to induce high levels of MD1 expression and complete rescue of muscle mass, specific force and resistance to eccentric contraction in *mdx* mice^20^,^22^,^23^. Intramuscular injection of a rAAV-Spc5.12-MD1 vector in a canine model of DMD resulted in sustained levels of MD1 expression in the injected muscles^24^. The golden retriever muscular dystrophy (GRMD) model is considered a highly valuable preclinical platform to test gene therapy strategies^25^,^26^. However, most of the published studies used immunosuppressive regimens, and were not designed to demonstrate functional improvements after treatment^27^,^28^,^29^,^30^,^31^,^32^.

Here, we show for the first time the long-term therapeutic potential of locoregional (LR) and systemic intravascular (IV) administration of rAAV2/8-Spc12-cMD1 vector in GRMD dogs, in the absence of any immunosuppression. Both procedures are well tolerated, and expression of the canine MD1 (cMD1) significantly reduces the physiological decline in muscle strength of treated limbs and stabilizes clinical parameters in the treated animals, in the absence of toxicity and of deleterious humoral or cell-mediated immune responses against the transgene product. Importantly, gene expression and clinical benefit are sustained over time, up to 24 months after vector injection. This study, carried out in a large animal model of DMD, paves the way to clinical translation of rAAV-MD1 gene therapy in DMD patients.

## Results

### Study design

We tested the administration of a rAAV2/8 vector encoding a sequence-optimized cMD1 cDNA driven by the synthetic muscle- and heart-specific Spc5.12 promoter (rAAV2/8-Spc5.12-cMD1) injected via LR or systemic IV routes in male GRMD dogs. The study design is summarized in Table 1. First, four GRMD dogs (3.5 to 4 months old) were injected in one forelimb via LR transvenous infusion as previously described^33^, using a single administration of 1 × 10^13^ vector genome (vg) kg^−1^ of the therapeutic vector. The contralateral untreated forelimb in the LR-treated dogs acted as controls, and three control GRMD dogs were also included and received the vehicle only (Table 1). Age-matched wild-type (WT) animals were also enrolled as additional controls for nuclear magnetic resonance (NMR) and muscle strength evaluations. We set the total injected volume at 20% of the limb volume to match the injection protocol recently reported in patients with neuromuscular diseases^34^,^35^. Injected dogs were followed for 3 months and then euthanised. In a second part of the study, eight GRMD dogs (2 to 2.5 months old) were treated systemically by peripheral vein injection with two different doses of the therapeutic vector: the first group of 5 dogs (IV-A) received a single dose of 1 × 10^14^ vg kg^−1^ of rAAV2/8-Spc5.12-cMD1 while the second group of 3 dogs (IV-B) received a single lower dose of 2 × 10^13^ vg kg^−1^ (Table 1). At the time of manuscript submission, we were able to follow 2 high-dose individuals (dogs IV1 and IV2) up to 2 years after vector injection and the 6 other individuals ∼8 months after therapy. Nine supplemental untreated GRMD dogs were also included and followed in parallel of these IV-treated dogs (Table 1), and supplemental healthy dogs were also used as controls. Importantly, no animal received immunosuppression at any stage, and they were randomized to the different groups (group IV-A, group IV-B or controls IV) with no phenotypic selection. It should be noted however that the appearance of severe muscle weakness, dysphagia and respiratory abnormalities before the age of 2 months (which occur in <5% of the global number of GRMD dogs of our colony) were considered as exclusion criteria for the study. Animals and biopsy samples were recoded in neutral fashion, and functional, histological and molecular analyses were performed in a blinded manner for both the LR and the systemic treatment studies.

### rAAV2/8-Spc5.12-cMD1 vector has good safety profile

Administration of rAAV2/8-Spc5.12-cMD1, both LR and systemic, was uneventful in all animals. Blood tests including electrolytes, kidney and liver function parameters and complete haematology counts remained unchanged in the hours, days and weeks after vector delivery (Supplementary Data 1 and 2). No evidence of vector-induced toxicity was observed in any of the treated dogs. Of note, dog IV5 suffered a treatment-unrelated accidental fracture of the left forelimb ∼2 months after rAAV injection. This fracture was surgically repaired and the dog entirely recovered his motor ability at <2 months after the accident. Overall, the rAAV2/8-Spc5.12-cMD1 showed an excellent safety profile in both LR and systemically treated animals at any injected dose.

### Efficient cMD1 expression after LR administration

**Figure 1:**
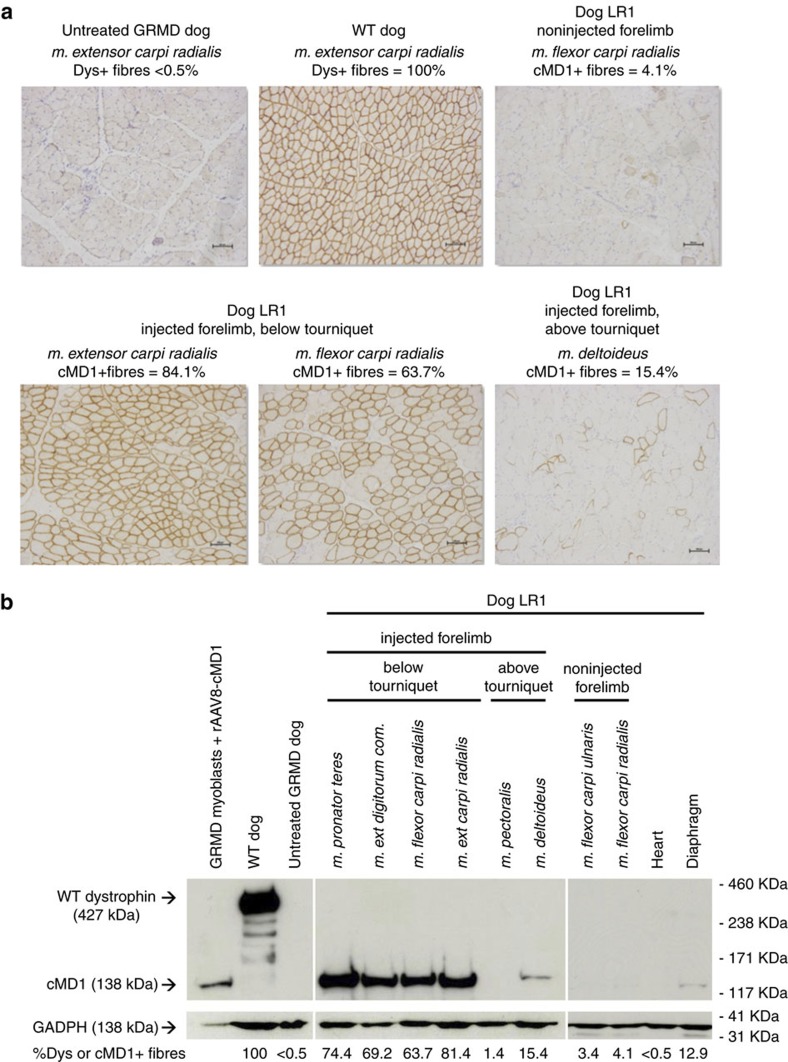
Analysis of cMD1 expression by immunostaining and western-blot in muscles of GRMD dogs injected with rAAV2/8-Spc512-cMD1 by the LR route. (**a**) Dystrophin immunostaining (NCL-DYSB) on transverse sections of muscle samples. Representative results are presented for healthy (WT) and untreated GRMD dogs and for four different muscles of dog LR1 sampled at the time of killing (non injected and injected forelimb, below and above the tourniquet). Scale bar, 100 μm. (**b**) Western-blot analysis of total proteins (50 μg) extracted from muscles samples. Representative results for the same muscles as in **a** are shown. GRMD myoblasts transduced with the rAAV2/8-Spc5.12-cMD1 vector were used as positive control. The blot was stained with MANEX-1011C to reveal the presence of the 427 kDa dystrophin protein (WT dog) and the 138 kDa cMD1 protein, with an anti-GAPDH antibody as a loading control. The level of cMD1-positive fibres detected by immunostaining from the same muscle samples are indicated under each panel.

The four LR-injected dogs were euthanised 3 months after vector injection. Skeletal muscles of each forelimb, as well as skeletal muscles at distance (total of 46 muscles) and several major organs were sampled after killing and used to evaluate cMD1 expression and vector genome copies per diploid genome (vg dg^−1^). cMD1 expression was evaluated by both immunoperoxidase staining and western blot. Figure 1a shows representative staining obtained in different muscles of dog LR1, sampled in the noninjected and injected forelimbs, below or above the tourniquet used to isolate the limb during the injection. MD1 was consistently expressed at high levels in muscle groups of the transduced forelimb, averaging 51%, 59%, 49% and 43% cMD1-positive fibres in dogs LR1 to LR4, respectively (Table 2). As observed in a previous study with a different rAAV2/8 vector^33^, shedding of the vector after release of the tourniquet also induced significant transduction in the contralateral limb and in distant muscle groups, with an average of 10%, 10%, 11% and 7% cMD1-positive fibres in dogs LR1 to LR4, respectively. In particular, significant transduction was observed in the diaphragm of dogs LR1 and LR3, with 13% and 18% of cMD1-positive fibres, respectively (Table 2). Immunolabelling data correlated proportionately with the detection of the ∼138 kDa cMD1 protein band by western-blot analysis (Fig. 1b) and of the cMD1 mRNA by reverse transcription quantitative PCR (RT-Q-PCR) in the same skeletal muscles samples (Supplementary Fig. 1). As shown in Supplementary Fig. 2, the percentage of cMD1-positive fibres and vector copy numbers, determined by Q-PCR analysis on the same muscle samples of the injected limbs, led to the direct correlation of ∼50% of cMD1-positive fibres being detected with the presence of 1 vg dg^−1^. Only low levels of cMD1 mRNA were detected in major organs (liver, spleen and lymph nodes) despite the presence of high vg dg^−1^ values, confirming the muscle-restricted activity of the Spc5.12 promoter (Supplementary Fig. 1).

### Expression of cMD1 improves muscle histopathology

**Figure 2:**
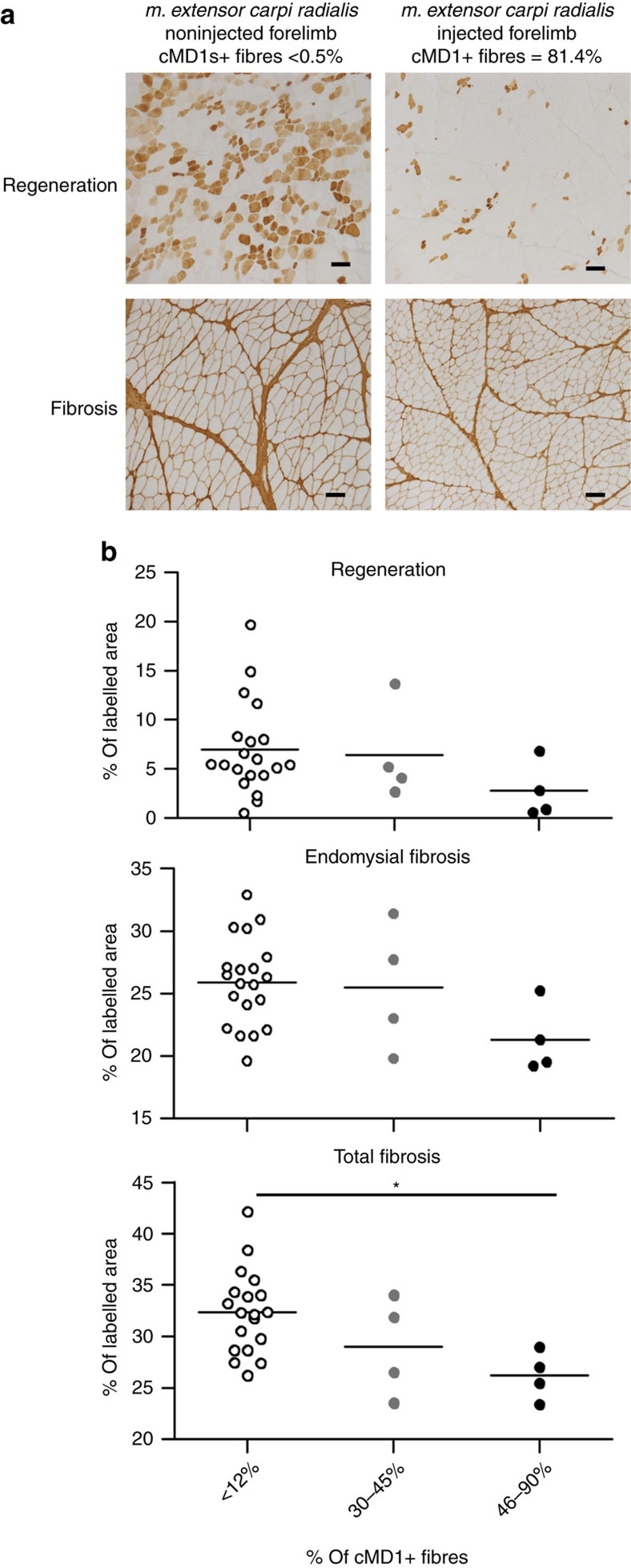
Improvement of pathological pattern in the cMD1-expressing muscles. After killing, two muscles (the *flexor carpi ulnaris* muscle and the *extensor carpi radialis* muscle) in each forelimb of each GRMD dog injected by the LR route either with the rAAV2/8-Spc5.12-cMD1 vector (*n*=4 dogs and 16 muscles analysed) or with buffer (*n*=3 dogs and 12 muscles analysed) were analysed. Myofibre regeneration was evaluated by immunohistochemical staining of myofibres using an antibody specific for developmental myosin heavy chain isoform. Total and endomysial fibrosis were evaluated by immunohistochemical detection of Collagen I. (**a**) Regeneration and fibrosis immunostaining on transverse sections of muscle samples. Representative results are presented for two muscles of dog LR1 (noninjected forelimb and injected forelimb). The levels of cMD1-positive fibres detected by immunostaining from the same muscle samples are indicated above each panel. Scale bar, 100 μm. (**b**) Regeneration, total fibrosis and endomysial fibrosis quantification in the total of 28 muscles analysed in the GRMD dogs injected by the LR route, either with the rAAV2/8-Spc5.12-cMD1 vector or with buffer was done using an automatic measurement of the percentage of the labelled area after selection of regions of interest. Analyses were done according to the percentage of cMD1-positive fibres of each muscle: <12% (*n*=20, empty symbols), between 30 and 45% (*n*=4, grey full symbols) and between 46 and 90% (*n*=4, black full symbols). Each point represents the data obtained in one muscle, and the horizontal bars represent the mean of the values obtained for each group *P<0.05 (nonparametric Kruskal–Wallis test with *post hoc* multiple comparison Dunn’s test).

Absence of dystrophin protein in both DMD patients and GRMD dogs results in cycles of muscle degeneration and regeneration that over time cause muscle tissue remodelling and replacement of muscle fibres by fibrotic tissue^36^,^37^. To investigate the effect of cMD1 expression on muscle pathology, we compared muscle regeneration and fibrosis in *extensor carpi radialis* and *flexor carpi ulnaris* muscles, sampled at the time of killing in both injected and noninjected forelimbs of each GRMD dog injected by the LR route either with the rAAV2/8-Spc5.12-cMD1 vector (*n*=4 dogs) or with buffer (*n*=3 dogs). In the four treated dogs, myofibre turnover (regeneration) and collagen deposition (fibrosis) were reduced in muscles of injected limbs compared with contralateral limbs (Fig. 2a). Since muscles of the noninjected limb were also transduced, although to a lesser extent, we correlated histological improvements with gene transfer efficiency, clustering the muscles in 3 groups based on the percentage of cMD1-positive fibres (that is, ≤12%, between 13 and 45% and between 46 and 90%). Muscles of treated limbs exhibiting >46% of cMD1-positive fibres showed a trend towards a decrease in myofibre regeneration and of endomysial fibrosis (Fig. 2b). In addition, a significant decrease of total fibrosis was observed in muscles harbouring >46% of cMD1-positive fibres compared with muscles with ≤12% of cMD1-positive fibres (*P*<0.05, Kruskal–Wallis test, Fig. 2b).

**Figure 3:**
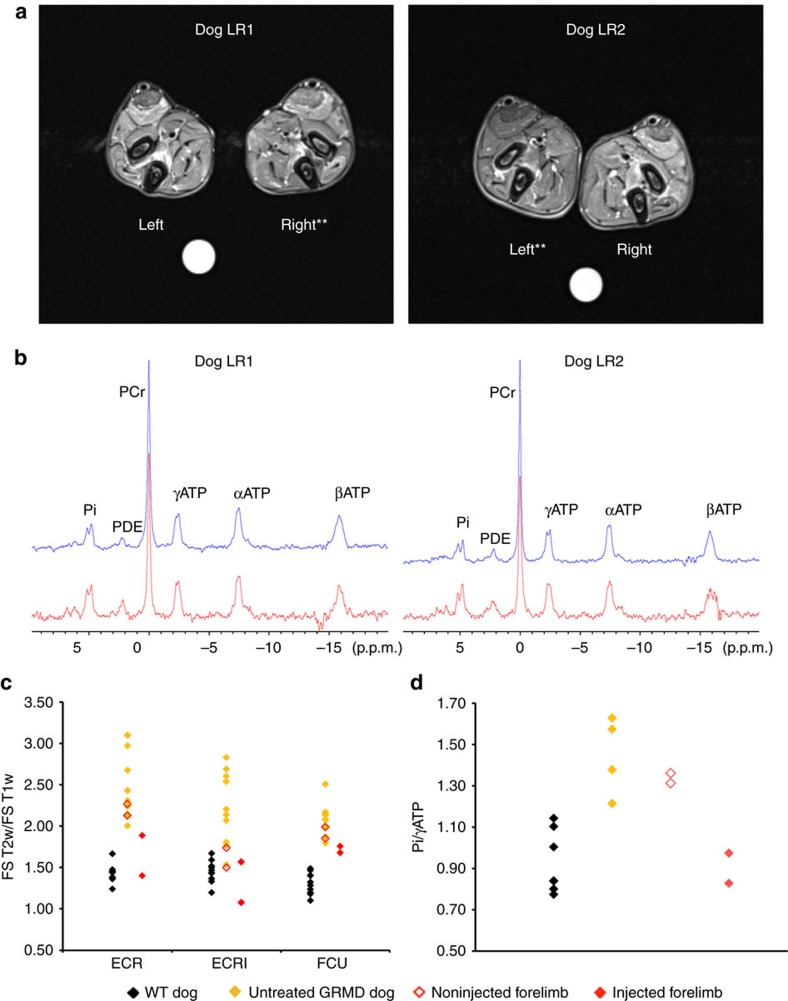
NMR imaging and spectroscopy analyses of muscles of GRMD dogs injected with rAAV2/8-Spc5.12-cMD1 by the LR route. (**a**) Representative example of transverse fat-saturated T2-weighted NMR image of the two forelimbs obtained in dog LR2. At 3 months after injection, signal muscle intensities were decreased and more homogeneous in the injected forelimb (**) compared with the noninjected one. (**b**) Representative example of ^31^P-NMR spectra of the injected (blue curve) and noninjected forelimb (red curve) of the same dog (LR2). Phosphocreatine (PCr) was increased and inorganic phosphates (Pi) and phosphodiesters (PDE) were decreased relative to ATP in the injected forelimb compared with the noninjected forelimb. (**c**) NMR imaging fat-saturated (FS) T2w/T1w muscle signal ratio obtained from three different muscles (ECR (*extensor carpi radialis brevis*), ECRl (*extensor carpi radialis longus*) and FCU (*flexor carpi ulnaris*)). The values of this index in the injected forelimb (red closed symbols) were decreased compared with the values obtained in the noninjected forelimb (open red symbols) or in untreated GRMD dogs (yellow symbols), and they were closer to the healthy dog (WT) indices (black symbols). (**d**) NMR spectroscopy Pi/γATP muscle signal ratios of the injected (red closed symbols) and noninjected forelimbs (open red symbols) as compared with untreated GRMD (yellow symbols) and healthy (WT) controls (black symbols).

Proton ^1^H-NMR imaging and phosphorous ^31^P-NMR spectroscopy analysis of treated and untreated forelimbs were performed just before killing in two dogs, LR1 and LR2. Dogs LR3 and LR4 were considered ineligible for the anaesthesia required for the exam due to a treatment-unrelated bronchopneumonia and were excluded from this analysis. The most relevant ^1^H-NMR imaging indices, relying on T2, T1 and proton density-weighted image signal intensities (T2w, T1w, proton density (PD))^38^, were calculated on three different muscles (*extensor carpi radialis brevis*, *extensor carpi radialis longus* and *flexor carpi ulnaris*). In ^31^P spectroscopy, signals were measured from the two *extensor carpi radialis* muscles. Visual assessment of ^1^H-NMR images in the muscles of the two analysed dogs showed altered magnetic resonance properties indicating a reduction of edema, inflammation, necrosis and cell damage. Indeed, muscle signals were less hyperintense on T2-weighted images and more homogeneous within and between muscles (Fig. 3a). We also observed a reversion in abnormal levels of phosphocreatine, inorganic phosphate and phosphodiesters in ^31^P-NMR spectra in the injected forelimbs of treated dogs, reflecting improved sarcolemmal membrane stability and muscle tissue metabolism^39^ (Fig. 3b). Finally, most imaging indices showed an improvement in the injected compared with the noninjected forelimbs, with a decrease of the fat-saturated T2w/T1w ^1^H-NMR imaging index, and the ^31^P-NMR spectroscopic Pi/γATP index to levels close to or within normal ranges of healthy dogs (Fig. 3c,d). Taken together, histology, NMR imaging and spectroscopy analysis confirmed a significant reduction of the dystrophic pathology in muscles of forelimbs injected with the rAAV2/8-Spc5.12-cMD1 vector.

### Expression of cMD1 improves muscle strength

**Figure 4:**
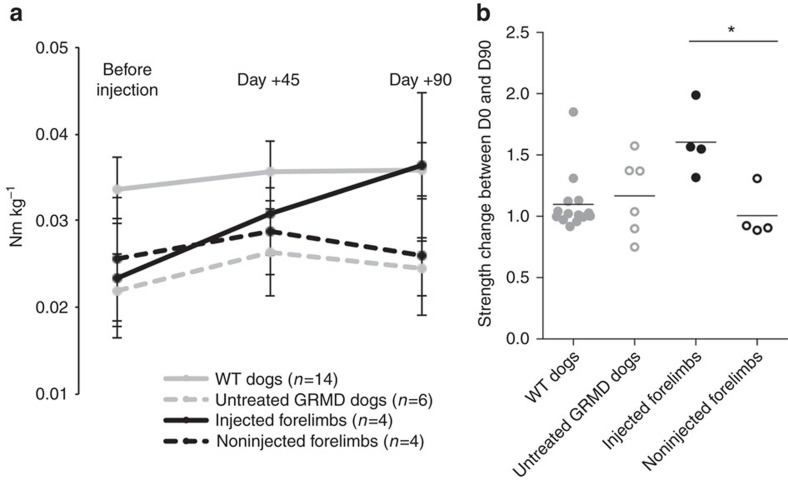
Evolution of the extension strength of the wrist in the forelimbs of GRMD dogs injected with rAAV2/8-Spc5.12-cMD1 by the LR route. The extension strength of the wrist of the forelimbs was measured using a specific torque measurement device. Three measurement sessions were performed all along the protocol: before injection, at day +45 and at day +90. (**a**) The evolution of the maximal torques, normalized by the animal weight, over the three different measurement sessions, was represented on each panel. Each point represents the mean value (±95% confidence interval) of the results obtained in forelimbs of healthy (WT) golden retriever dogs (*n*=14, grey line), in forelimbs of untreated GRMD dogs (*n*=6, grey dotted line), in the injected forelimb of rAAV2/8-Spc5.12-cMD1-treated GRMD dogs (*n*=4, dark line) and in the noninjected forelimb of the same dogs (*n*=4, dark dotted line). (**b**) Extension strength change between day 0 (before injection) and day +90. Each point represents the ratio between the maximal torque (normalized by the animal weight) obtained at day 0 and at day +90 in a same forelimb for healthy (WT) golden retriever dogs (*n*=14, grey full symbols), in untreated GRMD dogs (*n*=6, grey empty line), in the injected forelimb of rAAV2/8-Spc5.12-cMD1-treated GRMD dogs (*n*=4, black full symbols) and in the noninjected forelimb of the same dogs (*n*=4, black empty symbols). The horizontal bars represent the mean of the values obtained for each group. *P<0.05 (nonparametric Kruskal–Wallis test with post hoc multiple comparison Dunn’s test).

The effect of cMD1 expression on the functionality of the injected forelimb of all the LR-treated dogs was evaluated by measuring the extension strength of the wrist. We evaluated both forelimbs of the 4 injected GRMD dogs before LR treatment at 45 and at 90 days after buffer or rAAV2/8-Spc5.12-cMD1 injection. Seven age-matched WT golden retriever dogs were also evaluated in both forelimbs. At each time point, the maximal torque was measured and normalized by the animal’s weight. As shown in Fig. 4a, a clear difference was observed between the extension strength in forelimbs of WT and of untreated GRMD animals. Injection of rAAV2/8-Spc5.12-cMD1 significantly improved the muscle extension strength in the injected forelimbs compared with the noninjected ones as well as with those of untreated GRMD dogs, and normalized the values to the WT level at day +90. In the treated animals, the extension strength change between day 0 (before injection) and day +90 was significantly higher in the injected forelimb than in the noninjected forelimb (*P*<0.05, Kruskal–Wallis test, Fig. 4b). These results highlight the functionality of cMD1 in maintaining and eventually increasing muscle strength in dystrophin-deficient treated limbs.

### Body-wide expression of cMD1 improves pathology in GRMD dogs

**Figure 5:**
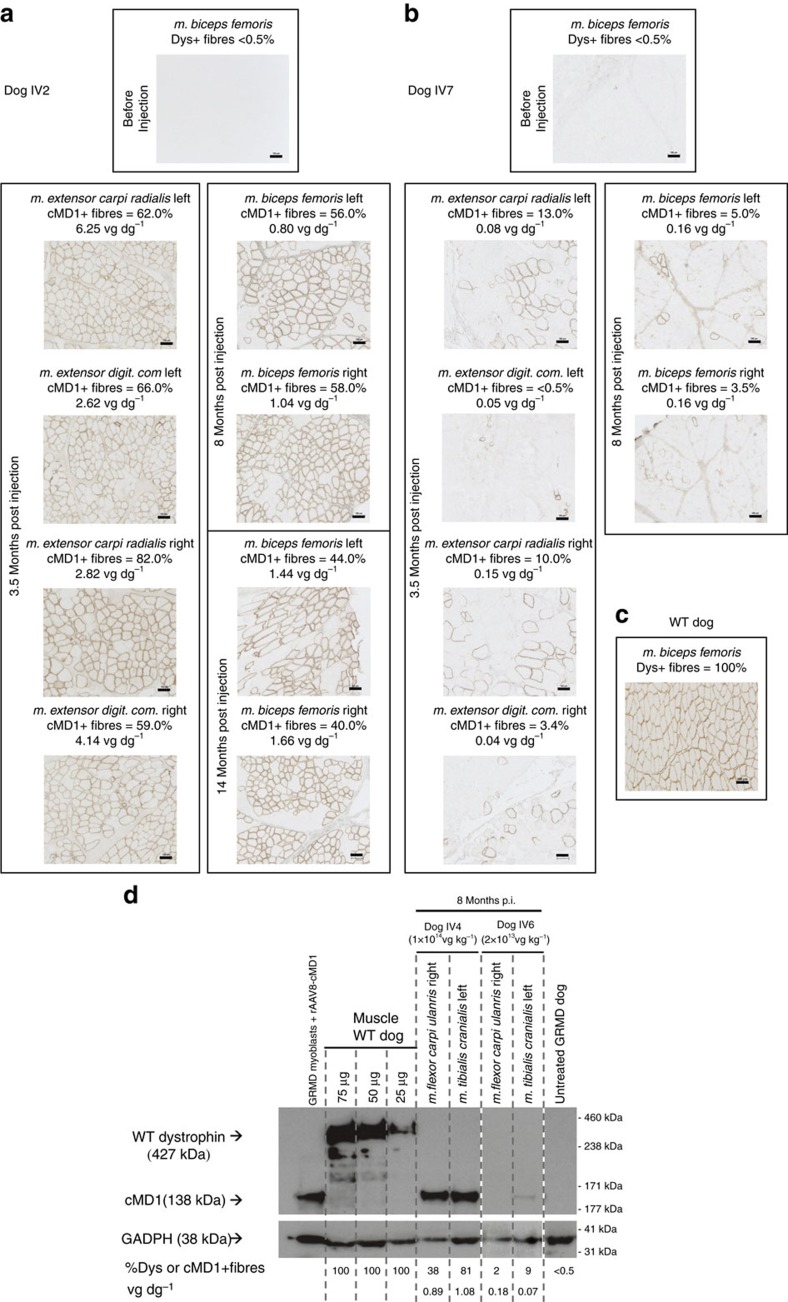
Analysis of cMD1 expression by immunostaining and western-blot in muscular biopsies of GRMD dogs injected with rAAV2/8-Spc512-cMD1 by the IV route. (**a**–**c**) Dystrophin immunostaining (NCL-DYSB) on transverse sections of muscle samples. Representative results are presented for two treated GRMD dogs injected with different doses of the rAAV2/8-Spc5.12-cMD1 vector by the IV route: dog IV2, injected with 1 × 10^14^ vg kg^−1^ (**a**) and dog IV7, injected with 2 × 10^13^ vg kg^−1^ (**b**). A healthy (WT) dog is also presented as control (**c**). For the treated dogs, different muscle samples were obtained after surgical biopsies performed before injection, at 3.5 months post injection, at 8 months post injection and at 14 months post injection (only for Dog IV2 for this latter time point). The level of cMD1-positive fibres is indicated above each panel, as well as the number of vector genomes per diploid genomes detected by qPCR in the same muscle sample. Scale bar, 100 μm. (**d**) Western blot analysis of total proteins (50 μg) extracted from muscles samples. Representative results are presented for two other treated GRMD dogs, injected with different doses of the rAAV2/8-Spc5.12-cMD1 vector by the IV route: dog IV4, injected with 1 × 10^14^ vg kg^−1^ and dog IV6, injected with 2 × 10^13^ vg kg^−1^. Then, 50 μg of total proteins extracted from GRMD myoblasts transduced with the rAAV2/8-Spc5.12-cMD1 vector were used as positive control, as well as 25 to 75 μg of total proteins extracted from a skeletal muscle of a WT dog. The blot was stained with MANEX-1011C to reveal the presence of the 427 kDa dystrophin protein (WT dog) and the 138 kDa cMD1 protein, with an anti-GAPDH antibody as a loading control. The level of cMD1-positive fibres detected by immunostaining as well as the number of vector genomes per diploid genomes detected by qPCR from the same muscle samples are indicated under each panel.

Prompted by the encouraging results obtained in the LR delivery study, we then systemically administered the rAAV2/8-Spc5.12-cMD1 vector by a single intravascular injection in two groups of GRMD dogs: the first (IV-A, high dose, *n*=5) received a dose of 1 × 10^14^ vg kg^−1^ and the second (IV-B, low dose, *n*=3) received a dose of 2 × 10^13^ vg kg^−1^. The age of the animals at treatment was ∼2 months (Table 1). We report here the ongoing follow-up of the IV-injected animals at more than 2 years post vector administration in two dogs (dogs IV1–2) and at ∼7 to 8 months post injection in six further dogs (dogs IV3–8). Surgical biopsies were performed in different skeletal muscles before the treatment, and at 3.5, 8 and 14 months after vector injection. Representative cMD1 immunostaining and western-blot of several skeletal muscle biopsy specimens obtained from IV-A- and IV-B-treated GRMD dogs are presented in Fig. 5. Levels of cMD1-positive fibres and vg dg^−1^ values are plotted in Supplementary Fig. 3. All dogs in the IV-A group (high dose) exhibited significant levels of cMD1-positive fibres (20 to 80%) and vector transduction levels between 1 and 6 vg dg^−1^ in the various muscular biopsies. As an example, dog IV2 showed in the different biopsied muscles an average of 67%, 57% and 40% cMD1-positive fibres, respectively at 3.5, 8 and 14 months post injection, with vector copy numbers around 4, 1 and 1 vg dg^−1^ in the same muscle samples (Fig. 5a and Supplementary Fig. 3). As muscles were biopsied at different time points, and as tissue transduction could be heterogeneous even within a same muscle, we cannot stringently compare these data overtime. However, a clear and statistically significant (*P*<0.001 or 0.01, Mann–-Whitney test) dose reduction effect was observed in dogs of the IV-B group (low dose) that showed on average <10% of cMD1-positive fibres and no more than 0.1 vg dg^−1^ observed in muscle biopsies obtained at 3.5 and 8 months post injection (Supplementary Fig. 3). Immunolabelling and vg dg^−1^ data correlated proportionately with the detection of the ∼138 kDa cMD1 protein band by western-blot analysis. Of note, the levels of cMD1 protein detected in some skeletal muscles of dogs of the IV-A group reached at least 50% of the normal level of native dystrophin found in WT dogs (Fig. 5b). Regeneration and total fibrosis were also evaluated in muscle biopsies obtained at 3.5 months post injection and showed a significant correlation with the levels of cMD1-positive fibres, as observed in the LR administration study (Supplementary Fig. 4).

Overall, these data indicate that systemic delivery of rAAV2/8-Spc5.12 results in generalized and stable skeletal muscle transduction and cMD1 expression, accompanied by a significant attenuation of the dystrophic pathology in GRMD dogs.

### Body-wide expression of cMD1 improves clinical phenotype

Long-term expression of cMD1 in the skeletal muscle of dogs in the IV-A (high dose) group correlated with a significant improvement in a range of clinical parameters compared with untreated GRMD dogs. These dogs remained ambulant and clinically robust with some displaying prolonged survival with good clinical status up to 2 years of age. Even dog IV-5, who suffered an accidental fracture of the left forelimb ∼2 months after rAAV injection, entirely recovered his motor ability <2 months after the accident. Supplementary Movie 1 shows representative dogs from each group, that is, untreated, IV-A and IV-B at various times after treatment.

**Figure 6:**
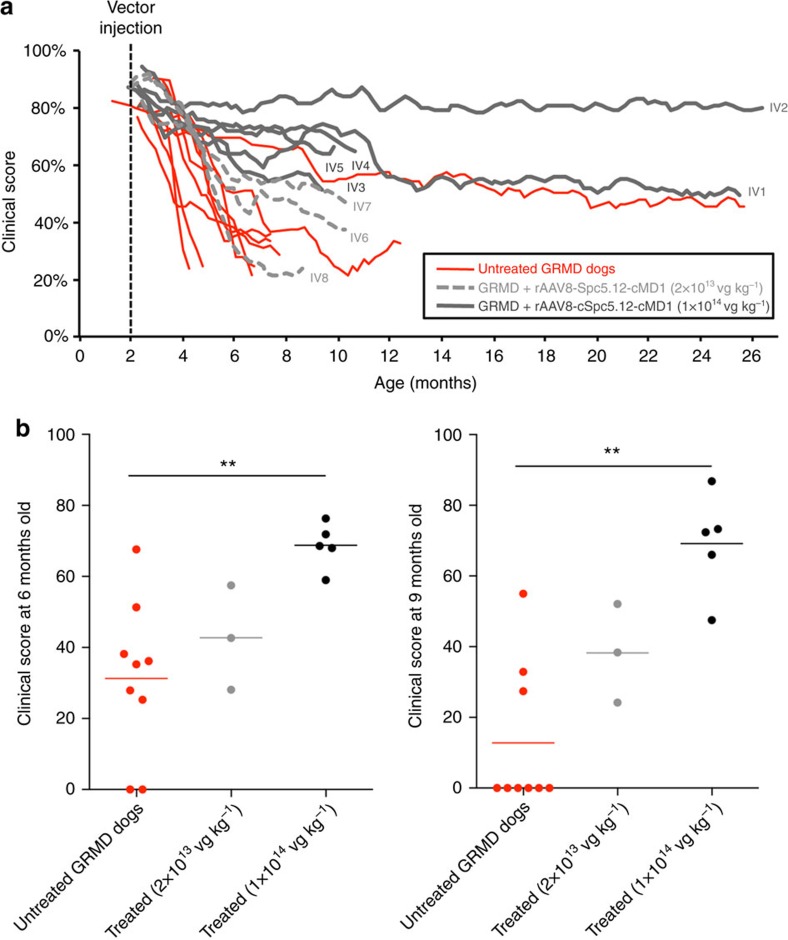
Improved clinical status of GRMD dogs injected with rAAV2/8-Spc5.12-cMD1 by the IV route. (**a**) The global clinical score was determined weekly and expressed as a percentage of a healthy dog score (100%) in untreated GRMD dogs (*n*=9, red lines) and GRMD dogs injected intravenously with rAAV2/8-Spc5.12-cMD1 at 2 × 10^13^ vg kg^−1^ (*n*=3, light grey dotted lines) or 1 × 10^14^ vg kg^−1^ (*n*=5, dark grey lines). For each dog, the line represents a tendency curve (mobile means order 3) built to show the score evolution. (**b**) The global clinical scores obtained in each dog at 6 months (left panel) and 9 months (right panel) of age were individually plotted for untreated GRMD dogs (*n*=9, red symbols), GRMD dogs injected intravenously with rAAV2/8-Spc5.12-cMD1 at 2 × 10^13^ vg kg^−1^ (*n*=3, grey symbols) or 1 × 10^14^ vg kg^−1^ (*n*=5, black symbols). Dead animals were reported with a clinical score at 0%. The horizontal bars represent the mean of the values obtained for each group. **P<0.01 (nonparametric Kruskal–Wallis test with *post hoc* multiple comparison Dunn’s test).

To quantify clinical improvement following systemic administration of rAAV2/8-Spc5.12-cMD1, a global clinical score was determined on a weekly basis by evaluating different parameters related to the GRMD pathology^40^. In the clinical scoring scheme, 100% represents a healthy WT golden retriever. As shown in Fig. 6a, untreated GRMD dogs and GRMD dogs in the low-dose treatment group rapidly deteriorated during the first 6- to 12-month period, at the end of which the majority (8 out of 9) of the untreated GRMD dogs were subjected to veterinarian-instructed killing. Indeed, even if a lifespan of ∼3 years was described in some GRMD colonies^26^, most untreated GRMD dogs of our colony show a rapid disease progression down to a clinical score of 30% at ∼1 year of age with death rapidly occurring after this point. A certain degree of variability among dogs may be observed, as previously reported^26^ and as observed in our study with in particular one untreated control animal (out of the 9 included in the study—dog IV C6) stabilized at a clinical score of ∼50% up to 26 months of age (Fig. 6a). Dogs in the high-dose group progressed much less rapidly than the majority of the untreated GRMD dogs, stabilizing in two cases at >50% (IV1) and >80% (IV2) at >2 years of age. Of note, even if dog IV1 exhibited the same clinical score (>50%) than one of the untreated GRMD dogs (dog IV C6), histological evaluations of muscle biopsies performed at 2 years of age in these 2 dogs showed a clear difference between these 2 animals in terms of pathological muscular pattern, with dystrophic features being numerous in the muscles of the untreated GRMD dog but highly reduced in the muscles of dog IV1 (Supplementary Fig. 5). Moreover, statistical evaluation of clinical scores at the 6 and 9 months time point showed significantly (*P*<0.01, Kruskal–Wallis test) higher scores in high-dose treated animals compared with untreated control animals (Fig. 6b). Notably, among the clinical parameters evaluated, the mean age of appearance of the characteristic dysfunctions in digestive (dysphagia) and respiratory (abdominal breathing) activities was substantially delayed in the high-dose group (Supplementary Table 1). Of note, none of our GRMD dogs (treated or untreated) carried the recently described mutation in the *Jagged1* gene (Supplementary Fig. 6) that can be responsible for a milder phenotype and a normal lifespan in GRMD dogs despite the complete absence of dystrophin^41^.

**Figure 7:**
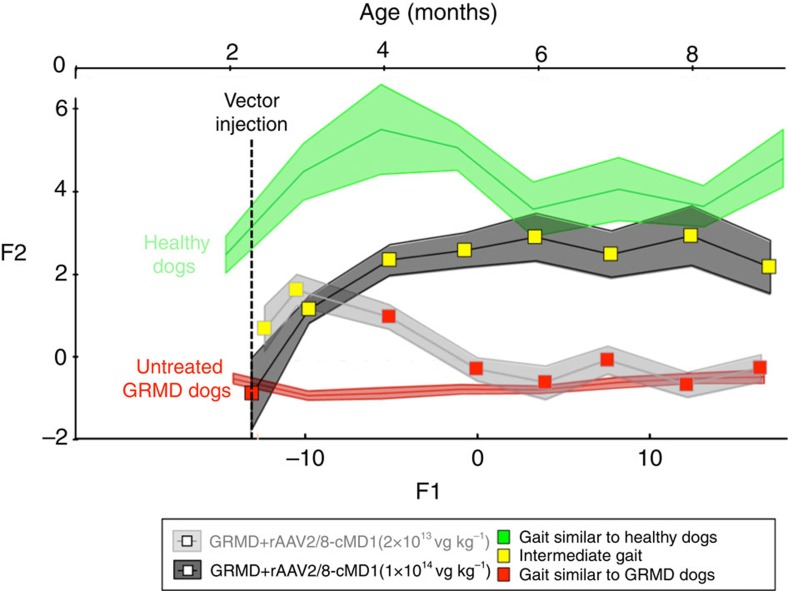
Improved gait quality of GRMD dogs injected with rAAV2/8-Spc5.12-cMD1 by the IV route. The global gait quality was determined twice a month using Locometrix and analysed by a discriminant analysis of seven accelerometric variables. The curves represent the evolution of the mean gait index with 95% confident intervals (shaded areas) in healthy dogs (*n*=9, including animals from retrospective cohorts, green curve), untreated GRMD dogs (*n*=25, including animals from retrospective cohorts, red curve) and GRMD dogs injected intravenously with rAAV2/8-Spc5.12-cMD1 at 2 × 10^13^ vg kg^−1^ (*n*=3, light grey curve) or 1 × 10^14^ vg kg^−1^ (*n*=5, dark grey curve). F1 and F2 represent the two axes used to plot data during discriminant analysis. An additional axis corresponding to the age in months was also calculated and represented. The discriminant analysis (see details in the Methods section) allows a statistical evaluation to test the probability that the gait of the treated animals was similar (*P*>0.95) to those of healthy dogs or of untreated GRMD dogs. The result of this statistical evaluation was represented as a colour code, using a green plot when the gait was found similar to those of healthy dogs, a red plot when similar to those of untreated GRMD dogs and yellow when the gait was considered as intermediate between healthy and untreated GRMD dogs.

In addition to clinical score parameters, we objectively evaluated the gait quality of the dogs on a bimonthly basis using the previously described Locometrix system^42^,^43^. For this study supplemental retrospective control data were assembled from groups of WT golden retrievers and untreated GRMD dogs. Figure 7 shows the evolution of the mean global gait index with 95% confidence intervals in healthy control dogs, untreated GRMD dogs and GRMD dogs injected systemically with rAAV2/8-Spc5.12-cMD1 at low dose or at high dose up to ∼7 months post injection. Dogs in the IV-A group showed a significantly higher gait index compared with untreated GRMD dogs, and an evolution towards a pattern very close to that of the wild-type golden retriever dogs. IV1 and IV2 dogs were evaluated until ≈1.5 year after injection and maintained a stable gait score (Supplementary Fig. 7). In line with the evolution of their clinical score, dogs of the IV-B group showed a transient improvement of the gait index that was not maintained at later time points (Fig. 7).

These data demonstrate that systemic delivery of rAAV2/8-Spc5.12-cMD1 substantially ameliorates the clinical manifestations and improves gait parameters of GRMD dogs.

### Evaluation of immune responses after vector delivery

We monitored humoral (presence of immunoglobulin G (IgG) antibodies in the serum) and cellular (specific interferon-γ (IFN-γ) secretion from peripheral blood mononuclear cells (PBMCs)) immune responses against the cMD1 protein in the GRMD dogs injected by LR or systemic IV route of administration (Table 3). We detected a transient humoral response against the cMD1 protein as soon as 1 or 2 months post injection in 3 out of 4 LR-injected and in 3 out of 8 IV-injected dogs, the latter all being injected with the high vector dose. In all dogs, anti-cMD1 IgG appear between 1 and 2 months after vector injection and then declined to undetectable levels at 8 months after vector injection (except for the IV4 dog for which IgG were still detectable at this time point—Table 3) without obvious or gross deleterious consequences. The transient nature of the anti-MD1 humoral immune response is shown as an example for the IV2 dog in Supplementary Fig. 8. T-cell responses directed against the cMD1 protein were assessed by IFN-γ enzyme-linked immunospot (ELISPOT) using stimulated PBMCs collected at different time points post injection. None of the injected dogs exhibited detectable levels of IFN-γ response above the threshold of positivity at any time point (Table 3). Dogs IV1 and IV2 maintained negative IgG and IFN-γ responses up to 2 years after injection (Table 3). We analysed anti-AAV8 capsid humoral and cellular immune responses in the four dogs injected via the LR route. As expected, and in line with previous studies^33^, we detected elevated and persistent IgG and neutralizing antibody levels to the AAV8 capsid in the serum of the four injected dogs (Supplementary Fig. 9), but no anti-capsid reactive T cells as measured by IFNγ ELISPOT (Supplementary Table 2).

Overall, these data suggest that LR or systemic administrations of rAAV2/8-Spc5.12-cMD1 vector elicit no detrimental humoral or cytotoxic immune responses in GRMD dogs despite the lack of immunosuppression.

## Discussion

Here, we show for the first time the effective, long-term and safe delivery of a microdystrophin transgene in the GRMD dog model of DMD by both LR and systemic administration of a rAAV2/8 vector and in the absence of preventive immunosuppression. In both studies, we utilized a sequence-optimized MD1 gene under the control of a skeletal muscle and heart-specific transcriptional regulatory element delivered in ‘GMP-like’ grade rAAV2/8 vectors. We initially tested the LR delivery of rAAV2/8-Spc5.12-cMD1 and observed high levels of cMD1+ fibres, averaging 50% in the muscles of the treated limb. These data correlated with cMD1 expression levels detected by western blot and RT-Q-PCR mRNA assays. Overall, high-level expression of the optimized cMD1 cassette combined with a LR route of delivery was associated with significant histological and functional amelioration in the injected limb, despite the reduced size of the protein and the absence of known functional domains, such as the neuronal nitric oxide synthase binding and relocalization domain^44^.

To our knowledge this is the first time a gene therapy based on rAAV-MD gene delivery by LR isolated limb perfusion and without immunosuppression has been performed in young adult GRMD dogs, showing clear prevention of degeneration/regeneration, fibrosis, magnetic resonance imaging and NMR changes and loss in muscle strength.

We and others previously showed the successful implementation of the LR delivery route by administration to GRMD dogs of rAAV2/8 vectors expressing exon-skipping antisense RNA sequences under the control of the U7snRNA promoter (rAAV2/8-U7snRNA-E6/E8)^33^,^45^. Interestingly, while both vectors were manufactured using an identical protocol, expression of a microdystrophin protein from the rAAV2/8-Spc5.12-cMD1 vector restored a higher number of phenotypically corrected fibres at comparable vg dg^−1^ values with respect to the exon-skipping vector that restores the synthesis of an almost full-length dystrophin protein. This may be explained by the stronger activity of the synthetic Spc5.12 promoter or by the overall higher efficiency of a gene replacement (one step event) compared with the splicing correction approach (two-step event).

We next evaluated the therapeutic effect of rAAV2/8-Spc5.12-cMD1 vector after systemic transvenous delivery in juvenile GRMD dogs, again in the absence of immunosuppression. We focused our investigations on relevant clinical outcomes, similarly to what would be applied in a phase I/II clinical trial in DMD patients. These included evaluation of the clinical status as well as of the gait of the animals, while biodistribution and expression of the transgene analyses were limited to iterative muscle biopsies over time. We observed a dose-dependent and significant stabilization, even improvement, of clinical parameters and of gait scores, with 2 × 10^13^ vg kg^−1^ being under-dosed, whereas 1 × 10^14^ vg kg^−1^ was effective, but still presenting heterogeneity among treated dogs. Nevertheless, this is the first demonstration of a long-term generalized clinical improvement of ‘*Jagged1*-negative’ GRMD dogs^41^ (up to 2 years of age for 2 dogs at the time of manuscript submission) after systemic administration of a rAAV2/8-MD vector. Unfortunately, since the emergence of cardiac pathology is very rare and with a late onset (over 1 year of age) in the untreated GRMD dogs of our colony, we have been unable to evaluate the impact of MD1 gene therapy on the cardiac function of the treated GRMD dogs by using conventional echocardiography, two-dimensional (2D) colour tissue Doppler imaging or even speckle tracking imaging (Supplementary Fig. 10). The recently described DMD^*mdx*^ rat model, which presents cardiac hypertrophy followed by a dilated cardiomyopathy similar to the one observed in DMD patients^46^, should allow a better evaluation of the impact of rAAV2/8-Spc5.12-MD1 treatment on the dystrophin-deficient cardiac pathology.

Only a handful of studies addressed so far the issue of body-wide systemic gene therapy in dystrophic dogs. A rAAV2/9 vector carrying a human sequence-optimized microdystrophin construct driven by the ubiquitous cytomegalovirus (CMV) promoter was injected intravenously in three 4-day-old dystrophic dog neonates^47^. In this study, the early inflammatory side effects observed in the pelvic region were likely linked to the use of the ubiquitous CMV promoter and/or to the expression of a non-species-specific human protein that may have induced a strong immune response in the muscles. No immune suppression was used in these dogs as animals treated soon after birth were expected to mount a less robust response due to the relatively immature nature of their immune system^48^. In another study^32^, dystrophic dog fetuses were transduced intra-amniotically to investigate the therapeutic effects of a rAAV vector expressing microdystrophin under conditions of immune tolerance. At 6 weeks after birth, rAAV2/9-CMV-microdystrophin was reinjected into the jugular vein of one of the dystrophic dog to induce systemic expression of MD. Gait and cardiac function improvement was observed in the treated dogs, suggesting that administration of a rAAV-microdystrophin vector after *in utero* tolerization could induce successful long-term transgene expression. A recent study^49^ in two 2-month-old DMD dogs used a tyrosine engineered modified rAAV9 capsid as the vehicle to deliver a canine sequence-optimized flag-tagged microdystrophin construct driven by a CMV promoter. Animals were euthanised at 4 months post injection, and sustained immunosuppression (cyclosporine A+mycophenolate mofetil) was applied. Although high doses (up to 5–6 × 10^14^ vg kg^−1^) were systemically injected, an average of only 25% of MD-positive fibres were found in this limited cohort and no functional or histopathological improvements were reported.

Unlike rodents, dog immunity shares many common features with its human counterpart with a full development before birth, although the maturity of the immune system completes after birth^50^. An important observation of our study, in view of clinical translation, was the lack of systemic adverse effects and anti-cMD1 T-cell immune responses at the highest, efficacious dose of cMD1 vector, associated with long-term persisting transgene expression despite the occasional and transient detection of anti-cMD1 IgGs in most of the dogs. Importantly, the transient humoral immune response was apparently without serious consequences in the transduced muscles or in peripheral organs. In fact, the detection of humoral immunity against a transgene product is not systematically associated with immunotoxicity in gene therapy protocols. The same type of non-deleterious humoral immune response has already been shown in haemophilia dogs following LR rAAV vector delivery without affecting factor IX transgene expression in animals^51^. This lack of deleterious adverse inflammatory and immunological reactions to the cMD1 transgene product may be due to several factors. Unlike intramuscular injection, LR and systemic transvenous infusion routes are associated with a lack of immunotoxicity as previously described for other transgenes^33^,^51^,^52^,^53^. It was also previously demonstrated in a mouse model of limb-girdle muscular dystrophy-2D that the use of a muscle-specific synthetic promoter, such as the Spc5.12 promoter^21^ used to restrict transgene expression in the present study, can lead to long-lasting transgene expression, whereas immune rejection was observed when the transgene is driven by the ubiquitous CMV promoter^54^. Furthermore, the presence of natural dystrophin ‘revertant fibres’ in GRMD dogs (as in the majority of DMD patients^55^) could be a powerful tolerogenic factor. Moreover, while we did observe a humoral (IgM, IgG and NAB) immune response to AAV8 capsid in the treated dogs, there was no detectable cellular immune response against proteins of the AAV8 capsid, in line with our previous studies^33^. The lack of detectable T-cell responses may be the result of rAAV2/8 vector produced in GMP (good manufacturing practices)-compliant baculovirus-insect Sf9 cell factories that may be effective in tackling adjuvant or proinflammatory effects of vector-associated or mammalian cell by-products observed in rAAV lots generated by standard co-transfection protocols in 293 cells^56^,^57^. However, it is important to note that in current and ongoing rAAV vector gene therapy trials in humans, low-level and variable transient hepatoxicity has been reported to be associated with cellular immune responses to AAV components and to be controlled by corticosteroid immunosuppression^58^. In the case of DMD, corticosteroid therapy has become the standard of care. It is thus likely that any future MD gene therapy clinical trial will recruit patients already taking and continuing to take corticosteroids, and thus already immunosuppressed. We would also point out that seropositivity to AAV8 arising from exposure to WT AAV8 virus or to a recombinant gene delivery vector will most likely preclude subjects from subsequent rAAV8-mediated gene therapy. Powerful physical (for example, plasmapheresis) and/or drug-induced immunosuppressive regimens are being investigated to potentially enable administration of rAAV gene therapy vectors in the presence of preexisting AAV antibodies^59^.

In conclusion, our data support the overall safety and therapeutic efficacy in GRMD dogs of both LR and systemic transvenous infusion of rAAV2/8-Spc512-cMD1, paving the way for human clinical trials for either upper limb or whole body delivery in a manner potentially therapeutic for any DMD patients irrespective of their mutation genotype.

## Methods

### Vector design and production

Our vector contained a species-specific codon and mRNA sequence-optimized canine microdystrophin cDNA, that is, cMD1: ΔR4–23/ΔCT, that was placed under the control of muscle and cardiac synthetic Spc5.12 promoter^24^. Two rAAV2/8-Spc5.12-cMD1 vector batches were produced using the Sf9/baculovirus expression vector system adapted for the rAAV8 (ref. 33). Briefly, Sf9 cells were cultured in serum-free insect cell medium (Sf-900 II SFM medium, Life Technologies) at 27 °C using 50 l of disposable bioreactors (Sartorius). After achieving the cell concentration of 1 million cells per ml, Rep2-Cap8 and ITR-Spc5.12-cMD1 baculoviruses were used for the co-transduction. Cells were left growing and then lysed to release vectors into the supernatant. The lysate was clarified on a 0.45 μm glass fibre frontal filter (Pall) and then purified using immunoaffinity chromatography column (AVB Sepharose HP, GE Healthcare). Finally, the vector was concentrated and formulated in Ringer-lactate solution (Baxter), sterile filtered, aliquoted and frozen at <−70 °C. Finally, several quality controls tests (physical titre, infectious titre, level of endotoxin, purity, sterility, pH, osmolality and *in vitro* functionality on GRMD myoblasts) were performed on each batch of vector before administration to dogs.

### Animals and vector delivery protocols

A total of 12 affected male GRMD dogs were injected with the rAAV2/8-Spc5.12-cMD1 vector, with 4 being injected via LR transvenous perfusion of one forelimb and 8 being injected via the IV route. In addition, 12 supplemental age matched male GRMD dogs were included as dystrophin-negative controls, with 3 being injected via LR transvenous perfusion of one forelimb with Ringer-lactate solution and 9 being untreated and followed in parallel of the IV-treated dogs (Table 1). Ten age-matched male healthy (that is, non dystrophic) golden retriever dogs were used as controls. At the time of manuscript submission, all GRMD dogs except dogs IV1 and IV2 have been euthanised by intravenous injection of pentobarbital sodium (Dolethal®, Vetoquinol) in accordance with approved protocols.

Dogs were all obtained from the Centre d’Elevage du Domaine des Souches (Mezilles, France) or from the Boisbonne Center for Gene Therapy (ONIRIS, Atlantic Gene Therapies, Nantes, France), two breeding centres of the same colony. They were handled and housed in the Boisbonne Center for Gene Therapy. The institutional animal care and use committee of the Région des Pays de la Loire (University of Angers, France) as well as the French Ministry for National Education, Higher Education and Research approved the protocols (authorization no. 2011.31 for LR-injected animals and authorization no. 00815.02 for IV-injected animals). The protocol was performed without any immunosuppression regimen.

For LR transvenous injection, AAV8 seronegative animals were injected at the age of 3–4 months. Dogs were initially anaesthetized with ketamine (Imalgene 1,000, Merial) and diazepam (Valium, Roche) and then maintained under an inhalational mixture of isoflurane (Vetflurane, Virbac) and oxygen. Morphine was used as analgesic. Cephalic vein of anaesthetized dogs was cannulated with a 20-gauge catheter (B-Braun) and the forelimb was exsanguinated using an Esmarch bandage (Microtek Medical) and applying a pneumatic tourniquet (Dessillon-Dutrillaux) that was placed above the elbow of the dog and inflated to 310 mm Hg to prevent blood circulation in the limb. After removal of the Esmarch bandage, the solution (rAAV diluted in Ringer-lactate solution or Ringer-lactate solution alone for the control limbs) was perfused. Dogs were injected with a volume corresponding to 20% of the limb volume at a fixed flow rate of 10 ml min^−1^ using a volumetric pump (Fresenius Vial). The venous pressure at the level of the catheter was monitored during the whole procedure. Once perfusion was complete, the tourniquet was left in place for 15 min and finally released.

For systemic administration, AAV8 seronegative animals were injected at the age of 2 months by direct IV injection in a cannulated cephalic vein at a fixed flow rate of 3 ml min^−1^. Prior injection, the rAAV vector was diluted in Ringer-lactate solution to obtain a fixed total volume corresponding to 5 ml of perfusate per kg of animal.

For surgical muscular biopsies, anaesthesia was induced with ketamine (Imalgene 1,000, Merial) in combination with diazepam (Valium, Roche) and was maintained using an inhalational mixture of isoflurane (Vetflurane, Virbac) and oxygen. Analgesia was performed with morphine. To limit discomfort of the animal due to preinjection surgical biopsy, meloxicam (Metacam, Boehringer-Ingelheim) was administered on the day of injection and for the following 2 days.

### Immunostaining of cMD1-positive fibres

Muscles samples were snap-frozen in isopentane cooled in liquid nitrogen and stored at <−70 °C until processing. Dystrophin-positive fibres were numbered in a blinded manner on serial transverse sections after immunohistochemical revelation of dystrophin using a mouse monoclonal anti-dystrophin antibody (Novocastra NCL-DYS-B, clone 34C5, 1:50, Leica). Goat anti-mouse biotinylated IgG (1:300, Dako) was used as secondary antibody, diluted in 5% dog serum in phosphate-buffered saline (PBS). The sections were then incubated with streptavidin/horseradish peroxidase (HRP) (1:300, Dako) and then revealed using 3,3′-diaminobenzidine (DAB, Dako). Although dystrophin-positive fibres in positive samples were scattered all along the muscle samples as individual or more often as clustered fibres, a minimum of 3 microscopic fields at intermediate magnification were randomly chosen to finally observe a minimum of 250 fibres (intraobserver variation coefficient was below 5%). All measurements were performed using Nikon’s NIS-Elements software (Nikon). Statistical analyses were performed using the nonparametric Mann–Whitney test. A difference was considered to be significant at *P<0.05, **P<0.01 or ***P<0.001.

### Western blot analysis

For each muscle sample, total proteins were extracted from snap-frozen muscle samples using RIPA buffer (Tris 10 mM pH 7.5; NaCl 150 mM; EDTA 1 mM; NP40 1%; sodium deoxycholate 0.5%; SDS 0.1%) containing protease inhibitor cocktail (Sigma-Aldrich). Protein extracts at 50 μg were loaded on a NuPAGE Novex 3–8% Tris Acetate gel and analysed using the NuPAGE large protein blotting kit (Thermo Fisher Scientific). Membranes were blocked in 5% skim milk, 1% NP40 (Sigma-Aldrich) in TBST (Tris-buffered saline, 0.1% Tween-20) and hybridized with an anti-dystrophin antibody specific for exons 10 and 11 of the dystrophin protein (1:100, MANEX 1011C, clone 4F9—monoclonal antibody obtained from the MDA monoclonal antibody resource—ref. 60) and with a secondary anti-mouse IgG HRP-conjugated antibody (1:2,000, Dako). For protein loading control, the same membrane was also hybridized with an anti-canine GAPDH antibody (1:10,000, Clinisciences) and with a secondary anti-goat IgG HRP-conjugated antibody (1:2,000, Dako). Immunoblots were visualized by ECL Chemiluminescent analysis system (Thermo Fisher Scientific). All uncropped western blots included on the study are reported in Supplementary Fig. 11.

### Vector biodistribution analysis

Vector copy numbers in the muscular biopsies were determined in a blinded manner. Genomic DNA was extracted using Gentra Puregene kit (Qiagen) and TissueLyser II (Qiagen). Q-PCR analyses were conducted on a StepOne Plus (Life Technologies) using 50 ng of gDNA in duplicate. Reactions were performed in a final volume of 25 μl containing template DNA, Premix Ex taq (Ozyme), 0.3 μl of ROX reference dye (Ozyme), 0.4 μmol l^−1^ of each primer (Thermo Fisher Scientific) and 0.2 μmol l^−1^ of Taqman probe (Thermo Fisher Scientific). Vector copy numbers were determined using primers and probe specifically designed to amplify the cMD1 transgene (forward: 5′-CCAACAAAGTGCCCTACTACATC-3′; reverse: 5′-GGTTGTGCTGGTCCAGGGCGT-3′; probe: 5′-FAM-CCGAGCTGTACCAGAGCCTGGCC-TAMRA-3′). Primers and probe designed to amplify the canine β-glucuronidase gene (forward: 5′-ACGCTGATTGCTCACACCAA-3′; reverse: 5′-CCCCAGGTCTGCTTCATAGTTG-3′; probe: 5′-FAM-CCCGGCCCGTGACCTTTGTGA-TAMRA-3′) were used to determine the endogenous gDNA copy numbers. For each sample, Ct values were compared with those of different dilutions of linearized standard plasmids (containing either the cMD1 expression cassettes or the canine β-glucuronidase gene). The sensitivity of our test was 0.002 vg dg^−1^. Statistical analyses were performed using a nonparametric Mann–Whitney test. A difference was considered to be significant at *P<0.05, **P<0.01 or ***P<0.001.

### cMD1 mRNA-level analysis

Total RNA was extracted from muscles with TRIzol reagent (Thermo Fisher Scientific) and treated with RNAse-free DNAse I from the TURBO DNA-free kit (Thermo Fisher Scientific) according to the manufacturer’s instructions. Then, 500ng of this RNA was reverse transcribed using random primers (Thermo Fisher Scientific) and M-MLV reverse transcriptase (Thermo Fisher Scientific). Q-PCR analysis was then performed in a blinded manner using the same cMD1-specific primers and probe than for the detection of transgenic DNA. As an internal control, RPL32 dog ribosomal RNA was used to normalize the mRNA concentration (forward: 5′-TGGTTACAGGAGCAACAAGAA-3′; reverse: 5′- GCACATCAGCAGCACTTCA-3′; probe: 5′-FAM-TGCTGCCCAGTGGCTTCTGG-TAMRA-3′). For each RNA sample, Ct values were compared with those obtained with different dilutions of standard plasmids (containing either the cMD1 expression cassette or the canine RPL32 dog ribosomal gene). Results were expressed in relative quantities (RQ): RQ=2^−ΔCt^=2^−(Ct target−Ct endogenous control)^. For each RNA sample, the absence of DNA contamination was also confirmed by analysis of ‘cDNA-liked samples’ obtained without addition of reverse transcriptase in the reaction mix.

### Histomorphological analysis

Regeneration was evaluated after immunohistochemical detection of myofibres with an antibody specific of a developmental Myosin Heavy Chain isoform (1:20, Novocastra NCL-MHCd, clone RNMy2/9D2, Leica). Goat anti-mouse biotinylated IgG and DAB were used to reveal the signal as for the dystrophin staining. The percentages of labelled areas were measured after manual threshold on all muscle cross-sections (reproducibility coefficient: 17%). Total and endomysial fibrosis were evaluated after immunohistochemical detection of Collagen I (1:500, clone I-8H5, MP Biomedicals). Again, goat anti-mouse biotinylated IgG and DAB were used to reveal the staining. The endomysial areas were selected by the operator, the threshold level was selected and an automatic measurement of the percentage of the labelled area was done. Twenty fields were randomly chosen to finally evaluate the level of endomysial fibrosis in each muscle (reproducibility coefficients: 1% with × 10 magnification for total fibrosis and 7% with × 20 magnification for endomysial fibrosis). All measurements were automatically performed and in a blinded manner using Nikon’s NIS-Elements software (Nikon). Statistical analyses were performed using the nonparametric Kruskal–Wallis test and *post hoc* multiple comparison using Dunn’s test. A difference was considered to be significant at *P<0.05, **P<0.01 or ***P<0.001.

### NMR imaging and spectroscopy analysis

This assessment was done in a blinded fashion, that is, the operator was not aware of the injected versus the noninjected forelimb. NMR imaging was performed at 3-tesla in a Siemens Magnetom Trio TIM imager/spectrometer (Siemens) in the injected forelimb and in the noninjected contralateral forelimb of each LR-injected GRMD dog. Ten significant quantitative indices were calculated from the ^1^H-NMR signal of three different muscles (*extensor carpi radialis brevis*, *extensor carpi radialis longus* and *flexor carpi ulnaris*). The most relevant NMR imaging indices relying on T2, T1 and proton density-weighted image signal intensities (T2w, T1w, PD), that is, the T2w/PD and T2w/T1w ratios, the T2w heterogeneity and the maximum relative enhancement after intravenous bolus injection of Gadolinium chelate, were analysed. All indices from the muscles of the injected forelimb were compared with those of the noninjected forelimb, and to reference data collected in untreated GRMD and healthy dogs. The ^31^P spectroscopy was realized at 4-tesla in a 46-cm free bore magnet (Magnex Scientific) interfaced to a Bruker Biospec spectrometer (Bruker Medical Gmbh), with a 2 cm diameter coil-collecting signal from the two *extensor carpi radialis muscles*. Signals were measured from the two *extensor carpi radialis muscles*. Phosphocreatine, the β-phosphate of ATP, phosphomono and phosphodiesters and two resonances of inorganic phosphate were measured on ^31^P spectra from which 7 ratios and the pH values for both inorganic phosphate resonances were calculated.

### Strength assessment

This assessment was performed in a blinded fashion, that is, the operator was not aware of the injected versus the noninjected forelimb, and using a specific torque measurement device built around interchangeable torquemeters with a nominal scale of either 2 or 20 Nm (Scaime)^33^. The flexion and extension strengths of the wrist of both forelimbs were measured for each LR-injected GRMD dog and for untreated GRMD and healthy control dogs. Dogs were maintained under anaesthesia using Propofol (Rapinovet, Schering-Plough) to limit peripheral muscle relaxation. Two thin insulated needles (28 G, TECA, Viasys Healthcare) were used to directly stimulate the nerve of the carpal flexors and extensors. Either the median and ulnar common nerve branches or the radial nerve were stimulated with trains generated during 500 ms at supramaximal intensity and various stimulation frequencies (5, 10, 20, 25, 50, 100, 133 and 200 Hz). Biphasic stimuli with a total duration of 1 ms were used and 30 s rest periods were waited between contractions. The maximal torque detected over all the elicited tetanic contractions was used to measure the maximal strength of the dogs for each muscle function (flexion and extension) and each side (injected and control). Three measurement sessions were performed all along the protocol. For each measurement session, the maximal extension torque was expressed related to the animal weight in nm kg^−1^. Statistical analyses were performed using the nonparametric Kruskal–Wallis test and *post hoc* multiple comparison using Dunn’s test. A difference was considered to be significant at *P<0.05, **P<0.01 or ***P<0.001.

### Clinical follow-up

The general clinical status of the systematically injected GRMD dogs was evaluated by a clinical grading done weekly after injection by the same doctor of veterinary medicine. Examination was blinded at the beginning of the study, up to when the difference between treated and untreated dogs became apparent. This evaluation includes 11 locomotion criteria and 6 items related to the general health status (including dysphagia, ptyalism, global activity and breathing)^40^. Each item was scored from 0 to 2, with 0 corresponding to the absence of symptoms and 2 to maximum severity. The global clinical score was expressed as the percentage of the maximum clinical score (defined as 100% for a healthy dog) and a tendency curve (mobile means order 3) was built to represent the clinical score evolution. The clinical score evolution obtained in our injected dogs was compared with the clinical score evolution of several nontreated GRMD dogs. Statistical analyses were performed using the nonparametric Kruskal–Wallis test and *post hoc* multiple comparison using Dunn’s test. A difference was considered to be significant at *P<0.05, **P<0.01 or ***P<0.001.

### DNA sequence analysis of the *Jagged1* gene

Genomic DNA obtained from muscle samples of all untreated GRMD dogs and of the 8 GRMD dogs injected by the intravenous route with the rAAV2/8-Spc5.12-cMD1 vector was extracted using Gentra Puregene kit (Qiagen) and TissueLyser II (Qiagen). Then, 100 ng of genomic DNA was amplified by PCR using GoTaq DNA polymerase (Promega) and the following primers, specific of the promoter region of the canine *Jagged1* gene: forward 5′-ACCCAACCTTTTCTGCACTC-3′ and reverse 5′-CATAGCCAAGGTCGAAGGAA-3′, with a 55 °C annealing temperature and 35 cycles. PCR products (253 bp) were migrated on an agarose gel, purified using Nucleospin Gel and PCR Clean-Up kit (Macherey Nagel) and finally sequenced on each strand by Beckman Coulter Genomics, with the same primers as those used for PCR amplification.

### Gait analysis

Gait characteristics were acquired in a blinded fashion twice a month in all systemically injected GRMD dogs using Locometrix, a three-dimensional (3D) accelerometric device composed of three orthogonally positioned accelerometers. This construction allows the recording of the accelerations along the dorsoventral, craniocaudal and mediolateral axes of the dogs. It has been shown that 3D accelerometry measurements allow quantitatively describing gait impairment and its progression in GRMD dogs^42^,^43^. In the present study, for each test, seven gait variables were calculated: the stride frequency; the stride regularity; the total power of accelerations; the relative components of the total power along the three axes of the space; and the stride length normalized by the height at withers. We established a new method using discriminant analysis and these seven variables plus the age of the dogs (in days) to evaluate dog gait. Briefly, we built a global gait index model using reference data collected during a previous 3D accelerometer study of disease progression (retrospective data)^42^,^43^ and during this study, with a total of 25 untreated GRMD and 9 normal dogs^61^. This discriminant analysis allows a statistical evaluation to test the probability that the gait of the treated animals was similar (*P*>0.95) to those of healthy dogs or of untreated GRMD dogs. When the gait was found to be different from both healthy and untreated GRMD dogs, it was considered as intermediate.

### Follow-up of the immune responses

The analysis of anti-cMD1 IgG was performed in a blinded manner by a western-blot protocol. For this study protein cellular extracts obtained from 293 cells not transfected or transfected by a pCMV-cMD1 plasmid were used instead of protein muscular extracts. Proteins were loaded on a NuPAGE Novex 3–8% Tris Acetate gel and analysed using the NuPAGE large protein blotting kit (Thermo Fisher Scientific) and then transferred to a Hybond ECL nitrocellulose membrane (Thermo Fisher Scientific). Membranes were blocked overnight and then incubated for 2 h at room temperature with sera from injected dogs (dilution 1:500). Peroxidase-conjugated rabbit anti-dog IgG antibody (1:5,000, Jackson ImmunoResearch) followed by enhanced chemiluminescence detection (Pierce) were used for detection. MANEX 1011C antibody (1:100, monoclonal antibody obtained from the MDA monoclonal antibody resource^60^) revealed with a peroxidase-conjugated goat anti-mouse IgG antibody (1:2,000, Dako) was used as a positive control. Uncropped western blots are reported in Supplementary Fig. 11.

The levels of circulating anti-AAV8 total IgG and IgM were determined using specific enzyme linked immunosorbent assays (ELISAs). The rAAV8 particles were diluted in coating buffer (0.1 M carbonate buffer, pH 9.5) to a final concentration of 2 × 10^10^ vg ml^−1^. Then, 50 μl was added to each well of a 96-well Nunc Maxisorp immunoplate (Thermo Fisher Scientific). At the same time, proteins corresponding to contaminants purified during the steps of rAAV production, but in the absence of viral particle formation, were diluted in the same buffer and seeded, in parallel, in different wells in the same immunoplate at a concentration of 3.4 μg ml^−1^. This amount of protein corresponds to an amount of contaminant protein equivalent to that seeded in the AAV wells and the signal obtained at the end corresponds to the nonspecific signal that was removed from the signal obtained with the same serum in the corresponding AAV wells. Plates were then incubated overnight at 4 °C. The next day, plates were washed three times with blocking buffer (6% nonfat milk buffer in PBS) and then blocked with blocking buffer for 2 h at room temperature. Plates were again washed three times with wash buffer (0.05% Tween-20 in PBS) and then incubated with heat-inactivated serum diluted from 1:3 to 1:7,290 (or more diluted if necessary) for 1 h at 37 °C. After three washes, purified antibodies specific for IgG or IgM (Sigma Aldrich) were added and incubated for 1 h at room temperature. After incubation the plates were washed three times with wash buffer. HRP-conjugated sheep anti-mouse antibody (GE Healthcare) was added and incubated for 1 h at room temperature. Last, plates were washed three times with wash buffer and revealed with tetramethylbenzidine substrate solution (BD Biosciences) for 30 min in the dark. The reaction was stopped with H_2_SO_4_ solution and measurements were made at 450 nm. The results are expressed as arbitrary optical density (OD) units using a colourimetric amplification system based on peroxidase. The AAV-specific signal was reported as the OD from AAV-coated ELISA after removal of the OD obtained on contaminant protein ELISA, a nonspecific signal (OD AAV-OD nonspecific signal).

The levels of circulating anti-AAV8 NAF were determined as follows. On day 1, 48-well plates were seeded with 5 × 10^4^ Huh7 cells per well for 24 h. On day 2, recombinant AAV8-CMV-Luciferase (AAV-CMV-Luc) was diluted in Dulbecco’s modified Eagle’s medium (Thermo Fisher Scientific) supplemented with 10% fetal calf serum (Hyclone) and incubated with a 10-fold dilution, and then twofold serial dilutions (1:2 to 1:400 or more diluted if necessary) of heat-inactivated serum samples for 1 h at 37 °C. Subsequently, the serum–vector mixtures corresponding to 1 × 10^5^ vg per cell were added to cells plated on day 1 and incubated in Dulbecco’s modified Eagle’s medium/10% fetal calf serum for 48 h at 37 °C and 5% CO_2_. Each mix was performed in duplicate. Cells were then washed in PBS and lysed for 10 min in 0.2% Triton lysis buffer at 4 °C. The lysate was transferred to 96-well plates and then the luciferase activity was read with a luminometer (VICTOR2, PerkinElmer Life Sciences). Transduction efficiency was measured as relative light units per second per well and normalized per amount of protein per well expressed as optical density. The neutralizing titre was reported as the highest serum dilution that inhibited the rAAV transduction by ≥50% compared with the control without serum and correlated with the amount of protein quantified in each well after cell lysis by the Bradford assay.

Cellular immune responses against cMD1 were evaluated in a blinded manner with an IFN-γ ELISPOT assay using frozen PBMCs and an overlapping peptide library covering the sequence of the cMD1 protein (Pepscreen, Sigma). The library included 238 synthetic peptides of 15 amino acids overlapping on 10 mers that were divided in 3 pools to stimulate thawed PBMCs. The threshold of positivity was determined as minimum of 50 spot-forming cells per 10^6^ cells and >to 3 times the number of spots recorded with nonactivated cells.

Cellular immune responses against AAV8 were also evaluated with an IFN-γ ELISPOT assay using frozen PBMCs incubated in the presence of lentiviral vectors encoding the AAV8 VP1 capsid protein. The functionality of the lentiviral vectors was checked by RT-PCR analysis on transduced human PBMCs. ELISPOT results were based on spot-forming units/10^6^ cells. Samples were considered positive if the number of spots was 1.5 times (cutoff based on mean ratio of negative donors±3 s.d.) larger than the corresponding control obtained from an empty lentiviral vector. Assays were scored if the number of spots under stimulation was >10 spots per 2 × 10^5^ cells or >10,000 spots per 10^6^ cells for the phorbol myristate acetate/ionomycin control.

### Assessment of cardiac function

Cardiac function of GRMD dogs injected with rAAV82/8-Spc5.12-cMD1 by the IV route was evaluated monthly using conventional echocardiography, 2D colour tissue Doppler imaging and speckle tracking imaging, a sensitive approach allowing the detection of contractility defects. Untreated GRMD dogs were used as references. Conventional echocardiography and 2D colour tissue Doppler imaging were performed on conscious dogs in standing position monitored with a continuous electrocardiography using a Vivid 7 ultrasound unit equipped with 5–7.5 and 2–5 MHz phased-array transducers (GE, Waukesha, WI, USA) according to the recommendations from the American College of Veterinary Internal Medicine^62^. All data were transferred for offline analysis using a specific software (Echo Pac 5.4, GE) by two examiners who were unaware of the clinical status of the dogs. Several parameters were measured for the assessment of myocardial contractility. For conventional parameters, left ventricular (LV) dimensions, posterior wall and interventricular septal wall thicknesses were measured and left ventricular fractional shortening and ejection fraction (Teichholz method) were calculated. Pulsed Doppler of the mitral valve inflow was used to measure the ratio of early to late diastolic flow velocity). Radial myocardial velocities were measured by tissue Doppler imaging at the LV posterior wall on a short-axis view at the level of the papillary muscles and at the basal portion of the LV septal and lateral walls on an apical four-chamber view. Global circumferential strain was obtained from averaged measures of segmental strains by speckle tacking imaging in each of six predefined segments on a short-axis view.

### Data availability

The data that support the findings of this study are available from the corresponding authors on reasonable request.

## Additional information

**How to cite this article:** Le Guiner, C. *et al*. Long-term microdystrophin gene therapy is effective in a canine model of Duchenne muscular dystrophy. *Nat. Commun.* **8,** 16105 doi: 10.1038/ncomms16105 (2017).

**Publisher’s note:** Springer Nature remains neutral with regard to jurisdictional claims in published maps and institutional affiliations.

## Supplementary Material

Supplementary Information

Supplementary Data 1

Supplementary Data 2

Supplementary Movie 1

## Figures and Tables

**Table 1:** Characteristics of the different GRMD dogs of this study.

**Group**	**Dog**	**Mode of delivery**	**Injected product and dose**	**Injected rAAV batch**	**Age at inclusion/ injection**	**Follow-up duration after inclusion/injection**
Group	LR1	Locoregional	rAAV2/8-Spc5.12-	A.12005.PSR	4 Months	3 Months
LR	LR2	transvenous	cMD1	A.12005.PSR	4 Months	3 Months
	LR3	perfusion	1 × 10^13^ vg kg^−1^	A.12005.PSR	3.5 Months	3 Months
	LR4			A.12005.PSR	3.5 Months	3 Months
						
Controls	LR C1	Locoregional	Buffer	NA	3.5 Months	3 Months
LR	LR C2	transvenous		NA	4 Months	3 Months
	LR C3	perfusion		NA	3.5 Months	3 Months
						
Group	IV1	Intravenous	rAAV2/8-Spc5.12-	A.12005.PSR	2 Months	24 Months
IV-A	IV2		cMD1	A.12005.PSR	2.5 Months	24 Months
	IV3		1 × 10^14^ vg kg^−1^	A.12005.PSR	2 Months	7.5 Months
	IV4			A.12005.PSR	2.5 Months	8.5 Months
	IV5*			A.12005.PSR	2 Months	8 Months
						
Group	IV6	Intravenous	rAAV2/8-Spc5.12-	A.12005.PSR	2 Months	8.5 Months
IV-B	IV7		cMD1	A.12005.PSR	2 Months	8.5 Months
	IV8		2 × 10^13^ vg kg^−1^	14D0332	2.5 Months	6.5 Months
						
Controls	IV C1	NA	NA	NA	1.5 Months	3 Months
IV	IV C2	(untreated)		NA	3 Months	4.5 Months
	IV C3			NA	3 Months	4.5 Months
	IV C4			NA	3.5 Months	9 Months
	IV C5			NA	3 Months	4 Months
	IV C6			NA	3 Months	23 Months
	IV C7			NA	2 Months	3 Months
	IV C8			NA	2.5 Months	4.5 Months
	IV C9			NA	2 Months	7 Months

NA, not applicable.

^*^At 2 months post injection, accidental fracture of the left forelimb.

**Table 2:** Levels of cMD1-positives fibres found after immunostaining analysis (NCL-DYSB) within the muscles of GRMD dogs injected by the LR route.

**Dog**	**Injected forelimb (*****n*****=13 muscles)**				**Noninjected forelimb (*****n*****=13 muscles)**	**Other muscles at distance (*****n*****=17 muscles)**	**Heart**	**Diaphragm**
	**Mean of cMD1+ fibres**	**CV**	**Mean of cMD1+ fibres for the group**	**CV for the group**	**Mean of cMD1+ fibres**	**Mean of cMD1+ fibres**	**Mean of cMD1+ fibres**	**Mean of cMD1+ fibres**
LR1	51%	54%	50%	47%	3%	10%	<0.5%	13%
LR2	59%	30%			1%	10%	<0.5%	<0.5%
LR3	49%	58%			3%	11%	<0.5%	18%
LR4	43%	49%			1%	7%	<0.5%	1%
LR C1	<0.5%	NA	<0.5%	NA	<0.5%	<0.5%	<0.5%	<0.5%
LR C2	<0.5%	NA			<0.5%	<0.5%	<0.5%	<0.5%
LR C3	<0.5%	NA			<0.5%	<0.5%	<0.5%	<0.5%

CV, coefficient of variation.

For the complete list of muscles and tissues sampled at the time of killing, see Supplementary Table 2 in ref. 33.

**Table 3:** Anti-cMD1-specific circulating IgG antibodies and anti-cMD1 specific IFN-γ secretion from PBMCs of GRMD dogs injected with rAAV2/8-Spc5.12-cMD1 by the LR or the IV route.

**Group**	**Dog**	**Circulating anti-cMD1 IgG**								**IFN-γ secretion by PBMCs with cMD1 peptides**																	
		**Bef. inj.**	**M +1**	**M +2**	**M +3**	**M +4**	**M +8**	**M +12**	**M +22**	**Bef. inj.**	**M +1**	**M +2**	**M +3**	**M +4**	**M +5**	**M +6**	**M +7**	**M +8**	**M +9**	**M +11**	**M +12**	**M +14**	**M +16**	**M +18**	**M +20**	**M +21**	**M +23**
Group LR	LR1	−	**+**	**+**	−	NA	NA	NA	NA	−	−	−	−	NA	NA	NA	NA	NA	NA	NA	NA	NA	NA	NA	NA	NA	NA
1 × 10^13^ vg kg^−1^	LR2	−	**+**	**+**	−	NA	NA	NA	NA	−	−	−	−	NA	NA	NA	NA	NA	NA	NA	NA	NA	NA	NA	NA	NA	NA
	LR3	−	**+**	−	−	NA	NA	NA	NA	−	−	−	−	NA	NA	NA	NA	NA	NA	NA	NA	NA	NA	NA	NA	NA	NA
	LR4	−	−	−	−	NA	NA	NA	NA	−	−	−	−	NA	NA	NA	NA	NA	NA	NA	NA	NA	NA	NA	NA	NA	NA
																											
Group IV-A	IV1	−	−	+	−	−	−	−	−	−	−	−	−	−	−	−	−	−	−	−	−	−	−	−	−	−	−
1 × 10^14^ vg kg^−1^	IV2	−	+	+	+	**+**	−	−	−	−	−	−	−	−	−	−	−	−	−	−	−	−	−	−	−	−	−
	IV3	−	−	−	−	−	−	NA	NA	−	−	−	−	−	−	−	−	−	NA	NA	NA	NA	NA	NA	NA	NA	NA
	IV4	−	+	+	+	+	+	NA	NA	−	−	−	−	−	−	−	−	−	NA	NA	NA	NA	NA	NA	NA	NA	NA
	IV5	−	−	−	−	−	−	NA	NA	−	−	−	−	−	−	−	−	−	NA	NA	NA	NA	NA	NA	NA	NA	NA
																											
Group IV-B	IV6	−	−	−	−	−	−	NA	NA	−	−	−	−	−	−	−	−	−	NA	NA	NA	NA	NA	NA	NA	NA	NA
2 × 10^13^ vg kg^−1^	IV7	−	−	−	−	−	−	NA	NA	−	−	−	−	−	−	−	−	−	NA	NA	NA	NA	NA	NA	NA	NA	NA
	IV8	−	−	−	−	−	−	NA	NA	−	−	−	−	−	−	−	−	−	NA	NA	NA	NA	NA	NA	NA	NA	NA

Bef. inj., before injection; M, month; +, detection of circulating anti-cMD1 IgG antibodies; −, no detection of circulating anti-cMD1 IgG antibodies or no detection of IFN-γ secretion by PBMCs; NA, not applicable.
