# Supplementary material for: Long-term microdystrophin gene therapy is effective in a canine model of Duchenne muscular dystrophy
Source: Nat Commun. 2017 Jul 25;8:16105. doi: 10.1038/ncomms16105 (PMC5537486; doi:10.1038/ncomms16105)
Supplement: Supplementary Information [file ncomms16105-s1.pdf]

Type of file: PDF
Size of file: 0 KB
Title of file for HTML: Supplementary Information
Description: Supplementary figures and supplementary tables.

Type of file: XLS
Size of file: 0 KB
Title of file for HTML: Supplementary Data 1
Description: Clinical blood biochemistry parameters measured before injection and at different time-points after vector injection in GRMD dogs treated with rAAV2/8-Spc5.12-cMD1 by the LR or the IV route.

Type of file: XLS
Size of file: 0 KB
Title of file for HTML: Supplementary Data 2
Description: Clinical haematology parameters measured before injection and at different time-points after vector injection in GRMD dogs treated with rAAV2/8-Spc5.12-cMD1 by the LR or the IV route.

Type of file: MP4
Size of file: 0 KB
Title of file for HTML: Supplementary Movie 1
Description: Improved clinical status of GRMD dogs injected with rAAV2/8-Spc5.12-cMD1 by the IV route: representative dogs from each group, i.e., untreated, IV-A and IV-B at various times after treatment.

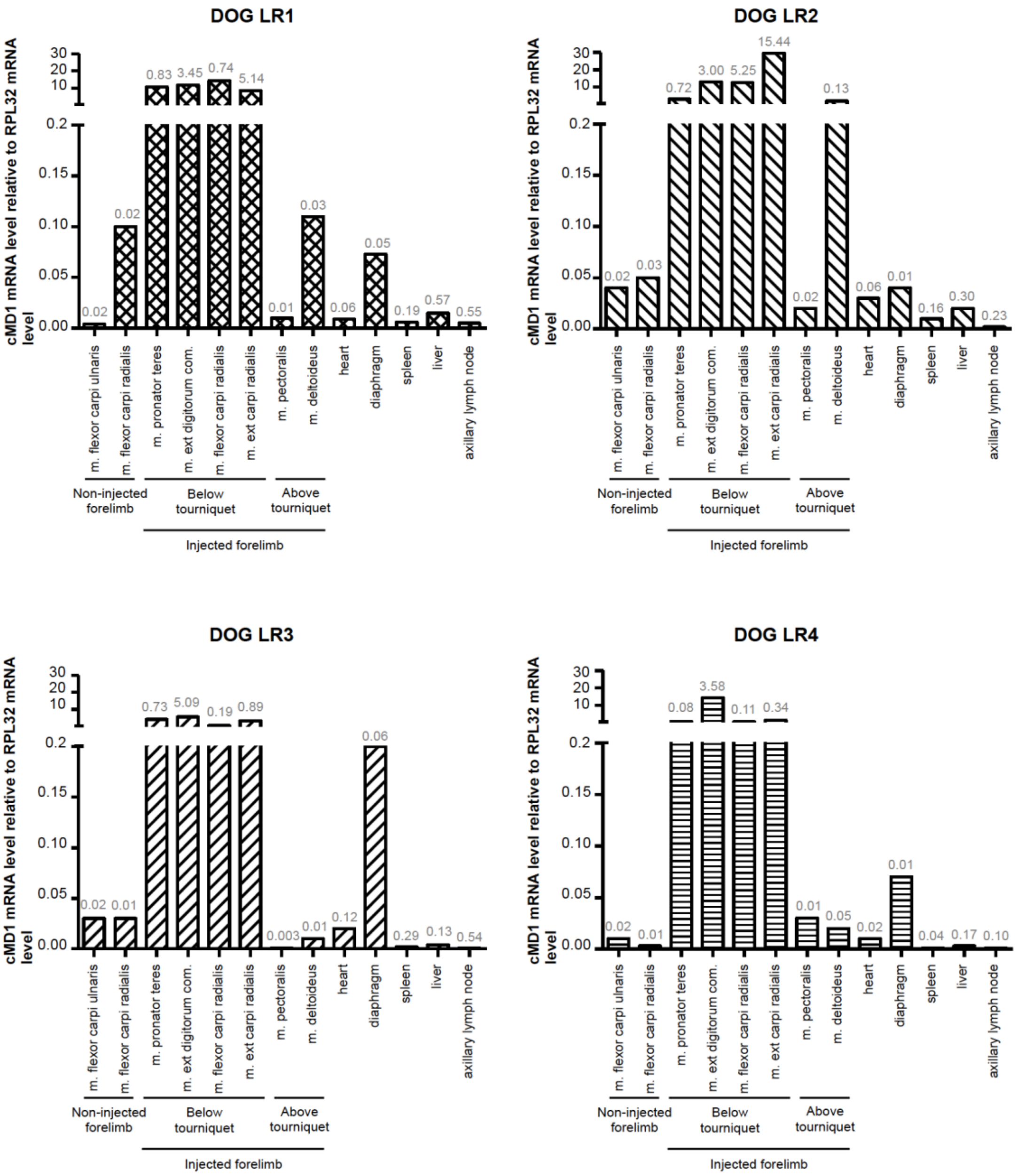

<u>Supplementary Figure 1:</u> **Levels of cMD1 mRNA in different tissues of the 4 GRMD dogs injected with rAAV2/8-Spc5.12-cMD1 by the LR route.** The levels of cMD1 mRNA in different tissues obtained at sacrifice were evaluated by RT-Q-PCR and normalized against the RPL32 dog ribosomal RNA. The copy numbers of vg/dg detected by Q-PCR on gDNA from the same tissue sample are indicated above each panel.

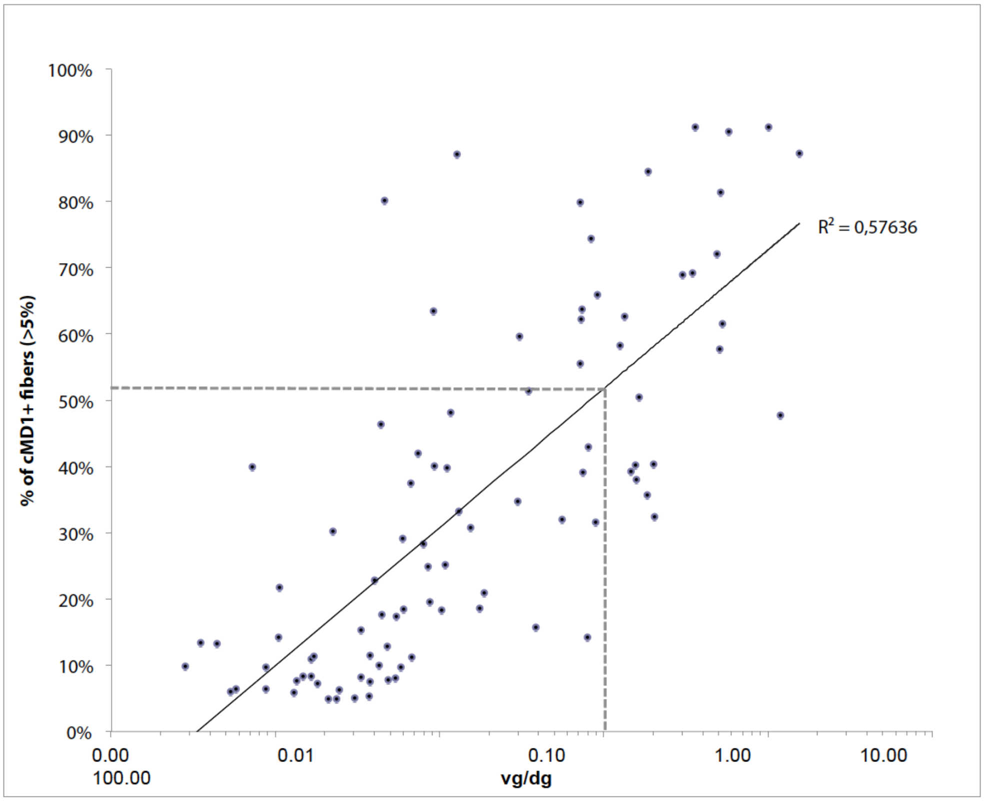

Supplementary Figure 2: Relationship between the percentage of cMD1-positive fibers and the vg/dg ratio in muscles of GRMD dogs injected with rAAV2/8-Spc512-cMD1 by the LR route. The percentages of cMD1-positive fibers obtained at sacrifice in each muscle of the injected forelimbs were correlated with the number of vg/dg in the same muscle. Muscles expressing more than 5% of cMD1-positive fibers were included in the analysis. The cumulative data displayed a linear regression, with a correlation coefficient (R^2) of 0.576 (for n=92 muscles analyzed). This correlation indicates that 1 vg/dg potentially translates to ~50% cMD1-positive fibers.

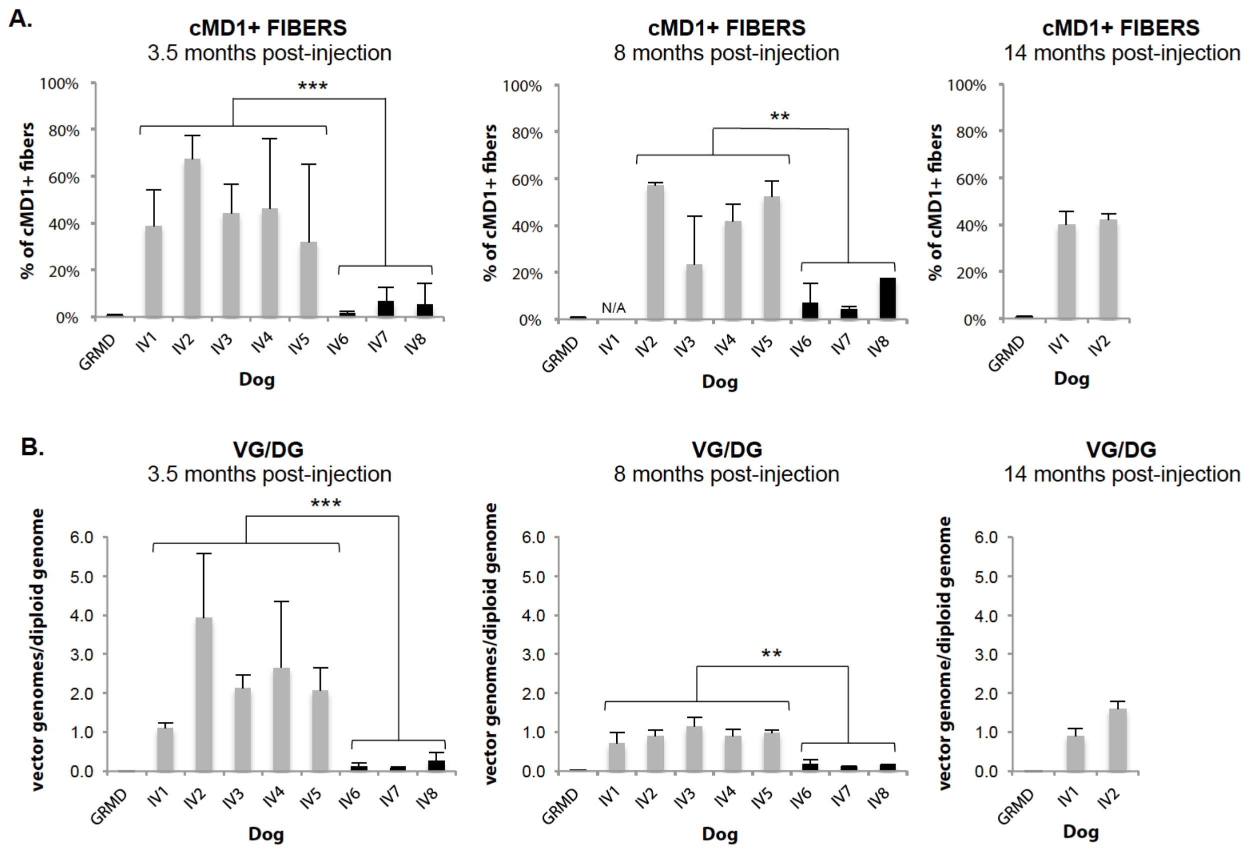

<u>Supplementary Figure 3:</u> Long-term cMD1 expression and vg/dg detection in muscles of GRMD dogs injected with rAAV2/8-Spc5.12-cMD1 by the IV route. The levels of cMD1-positive fibers detected by immunostaining (NCL-DYSB) **(A)**, and levels of vg/dg **(B)** were evaluated in the same surgical muscular biopsies obtained at 3.5 months post-injection, 8 months post-injection and 14 months post-injection in each GRMD dog injected with rAAV2/8-Spc5.12-cMD1 by the IV route. Each bar represents the mean (with standard deviation) of the results obtained in each dog. 4 muscles (*extensor carpi radialis,* left and right *and extensor digitorum communis*, left and right) were analyzed at 3.5 months post-injection and the 2 same muscles (*biceps femoris,* left and right) were analyzed at ~8 months (except 6.5 months for Dog IV8) and

at 14 months post-injection. Black bars were used for the dogs injected with $1x10^{14}$ vg/kg and grey bars were used for the dogs injected with $2x10^{13}$ vg/kg. GRMD = results obtained in a control non-injected GRMD dog. N/A = sample not available for analysis. *** $p<0.001$ and ** $p<0.01$ (non-parametric Mann-Whitney test).

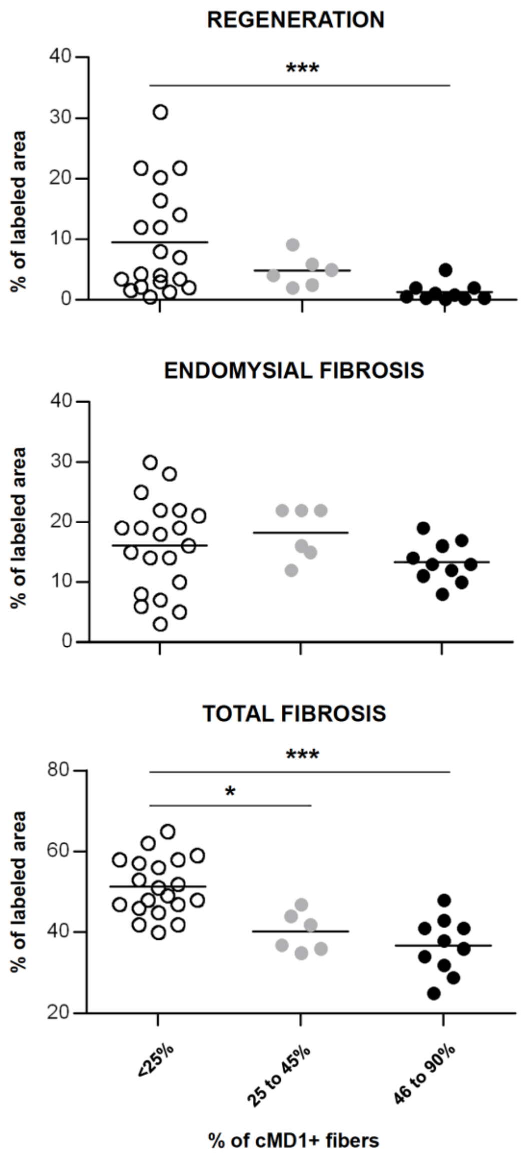

<u>Supplementary Figure 4</u>: **Improvement of pathological pattern in the cMD1-expressing muscles obtained at 3.5 months post-injection in GRMD dog injected by the IV route with the rAAV2/8-Spc5.12-cMD1 vector.** At 3.5 months post-injection, two muscles (the *flexor carpi ulnaris* muscle and the *extensor carpi radialis* muscle) in each forelimb of each GRMD dog injected by the IV route with the rAAV2/8-Spc5.12-cMD1 vector, as well as one untreated GRMD dog, were analyzed (n=9 dogs and 36 muscles analyzed). Myofiber regeneration was

evaluated by immunohistochemical staining of myotubes using an antibody specific for developmental myosin heavy chain isoform. Total and endomysial fibrosis were evaluated by immunohistochemical detection of Collagen I. Quantification was done using an automatic measurement of the percentage of the labeled area after selection of regions of interest. Analyses were done according to the percentage of cMD1-positive fibers of each muscle: <25% (n=20, empty symbols), between 25 and 45% (n=6, grey full symbols) and between 46 and 90 % (n=10, black full symbols). Each point represents the data obtained in one muscle, and the horizontal bars represent the mean of the values obtained for each group * $p<0.05$ and *** $p<0.001$ (non-parametric Kruskal-Wallis test with post-hoc multiple comparison Dunn test).

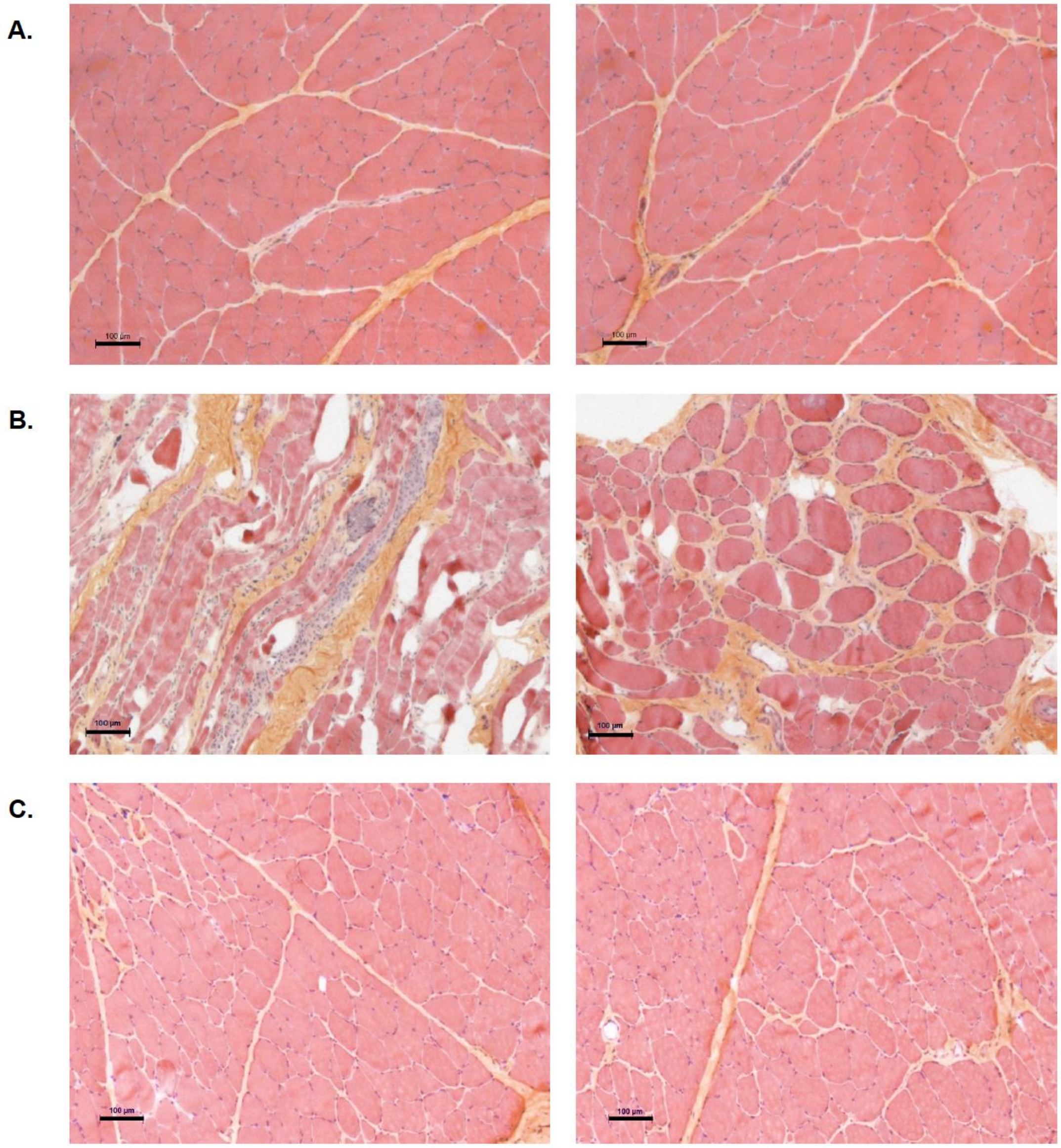

<u>Supplementary Figure 5:</u> Muscle histopathology (classical HES staining) in biceps femoris (right and left) of different dogs at 2 years of age.

(A) Representative healthy control dog exhibiting a clinical score of 100%

(B) The untreated GRMD dog IV C6 exhibiting a clinical score of 50%

(C) Dog IV-1 exhibiting a clinical score of 50%

Scale bar = 100µm.

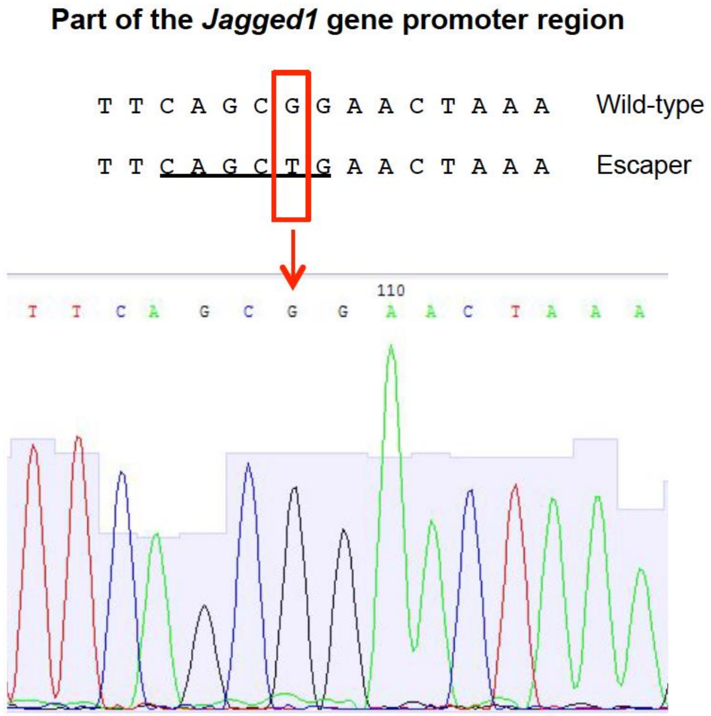

<u>**Supplementary Figure 6:**</u> **DNA sequence analysis of a part of the promoter region of the *Jagged1* gene in skeletal muscles of GRMD dogs.** A G>T change in this region creates a novel myogenin binding site (underlined sequence) in the promoter region of the *Jagged1* gene, increasing its expression in skeletal muscles. Overexpression of Jagged1 in GRMD dogs can be responsible for a milder phenotype and a normal lifespan despite the complete absence of dystrophin [41]. Such GRMD dogs are called "Escaper". Results of DNA sequence analysis obtained in Dog IV 4 are presented here as example and clearly show a "wild-type" genotype. Similar results were obtained in all GRMD dogs of this study (untreated or injected by the IV route with the rAAV2/8-Spc5.12-cMD1 vector).

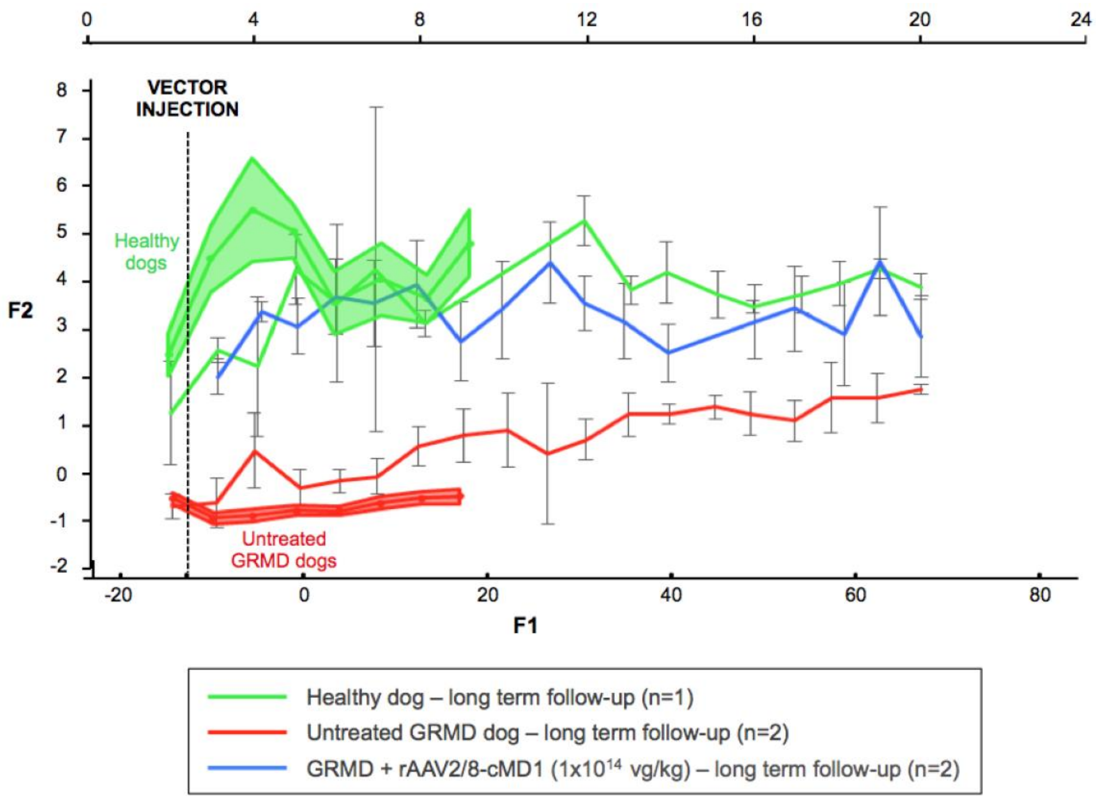

<u>Supplementary Figure 7:</u> **Long-term improved gait quality of GRMD dogs injected with rAAV2/8-Spc5.12-cMD1 by the IV route.** The global gait quality was determined twice a month using Locometrix® and analyzed by a discriminant analysis of 7 accelerometric variables. F1 and F2 represent the two axes used to plot data during discriminant analysis. An additional axis corresponding to the age in months was also calculated and represented. The curves with the shaded areas represent the evolution of the mean gait index with 95% confident intervals in 9 healthy dogs (green curve) and 25 untreated GRMD dogs (red curve) followed during 8 months and already showed on Figure 7. Among these animals (including some from animals from retrospective cohorts), some individuals were followed until 20 months of age and plotted on this graph as lines representing mean with 95% confident intervals: one healthy dog (green line), two untreated GRMD dogs (red line) and two GRMD dogs injected intravenously with rAAV2/8-Spc5.12-cMD1 at $1x10^{14}$ vg/kg (Dog IV1 and Dog IV2, blue line).

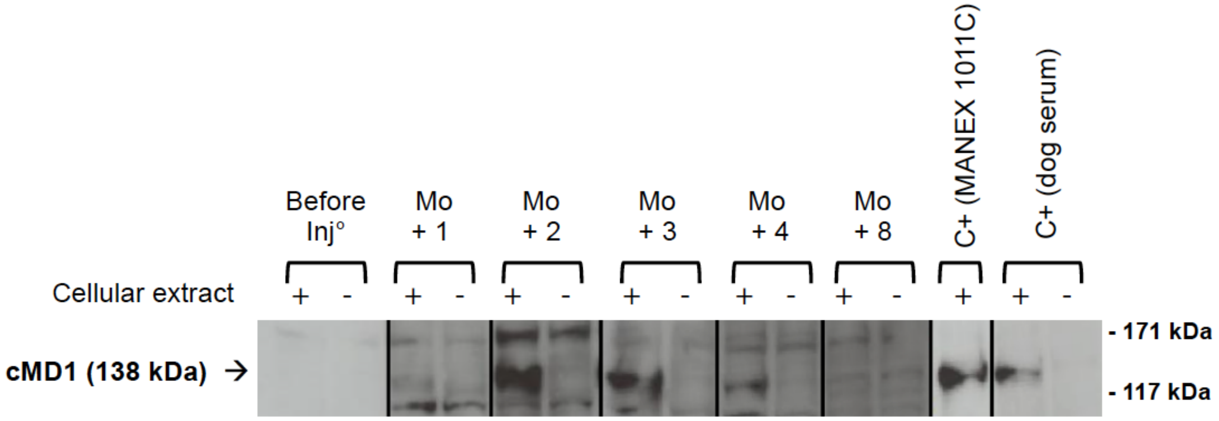

Supplementary Figure 8: Detection of anti-dystrophin IgG antibodies by Western-Blot in injected dog sera. Results obtained in Dog IV2 are presented here as example. The reactivity of each serum was tested on cellular extracts of 293 HEK cells transfected or not with a plasmid encoding the cMD1 protein, in order to assess specificity of potential detected cMD1 IgG antibodies. For each dog, sera before injection (Day 0) and after injection (here +1, +2, +3, +4 and +8 months) have been tested at the dilution 1/500. Positive controls consisted in the anti-dystrophin antibody MANEX 1011C (MDA Monoclonal Antibody Resource), and a positive canine serum (C+) from a GRMD dog immunized against dystrophin. High molecular weight protein standard was used to detect dystrophin band (138 kDa).

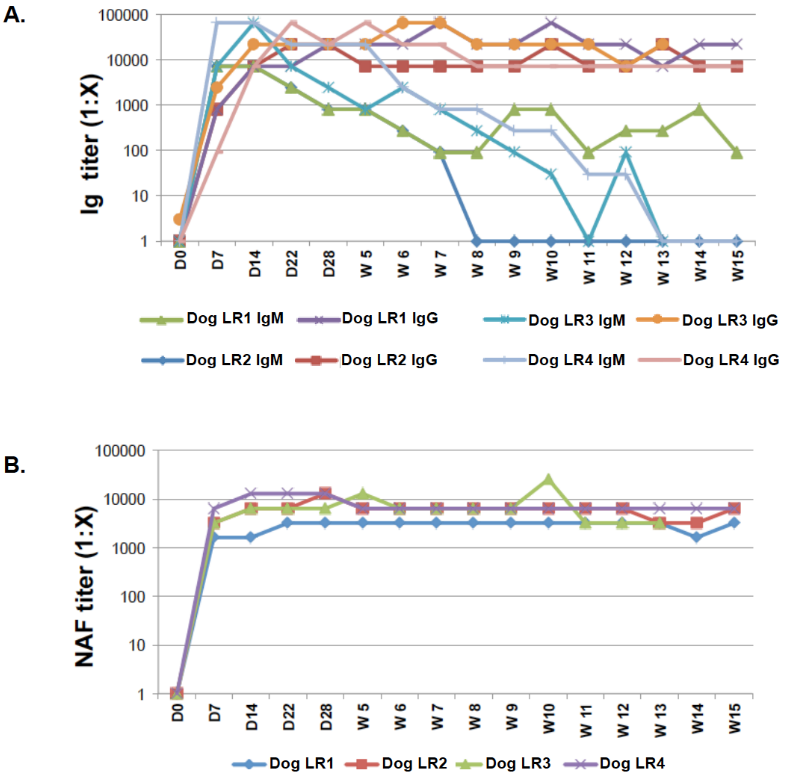

<u>Supplementary Figure 9:</u> Titers of anti-AAV8 IgG, IgM and NAF in the sera of GRMD dogs injected with rAAV2/8-Spc5.12-cMD1 by the LR route.

(A) Results of ELISA assays measuring specific IgG or IgM specific to AAV8 at different time points post-injection (D=day; W=weeks) in sera of dogs LR1 to LR4. Sera were considered positive for anti-AAV8 specific IgG or IgM when, at dilution ≥1:3, OD signal was ≥ 0.4 or 0.6 respectively (cut-off based on mean OD of 84 or 56 negative dogs + 3 SD, respectively). Final results were reported as anti-AAV8 antibody dilution (titer).

(B) NAF titers specific to AAV serotype 8 at different time points post-injection (D=day; W=weeks) in sera of dogs LR1 to LR4. Sera were considered positive for neutralizing capacity when a 1:2 dilution of serum inhibited vector transduction by 50% or more. The neutralizing titer was reported as the highest serum dilution that inhibited the rAAV transduction by ≥ 50% compared with the control without serum.

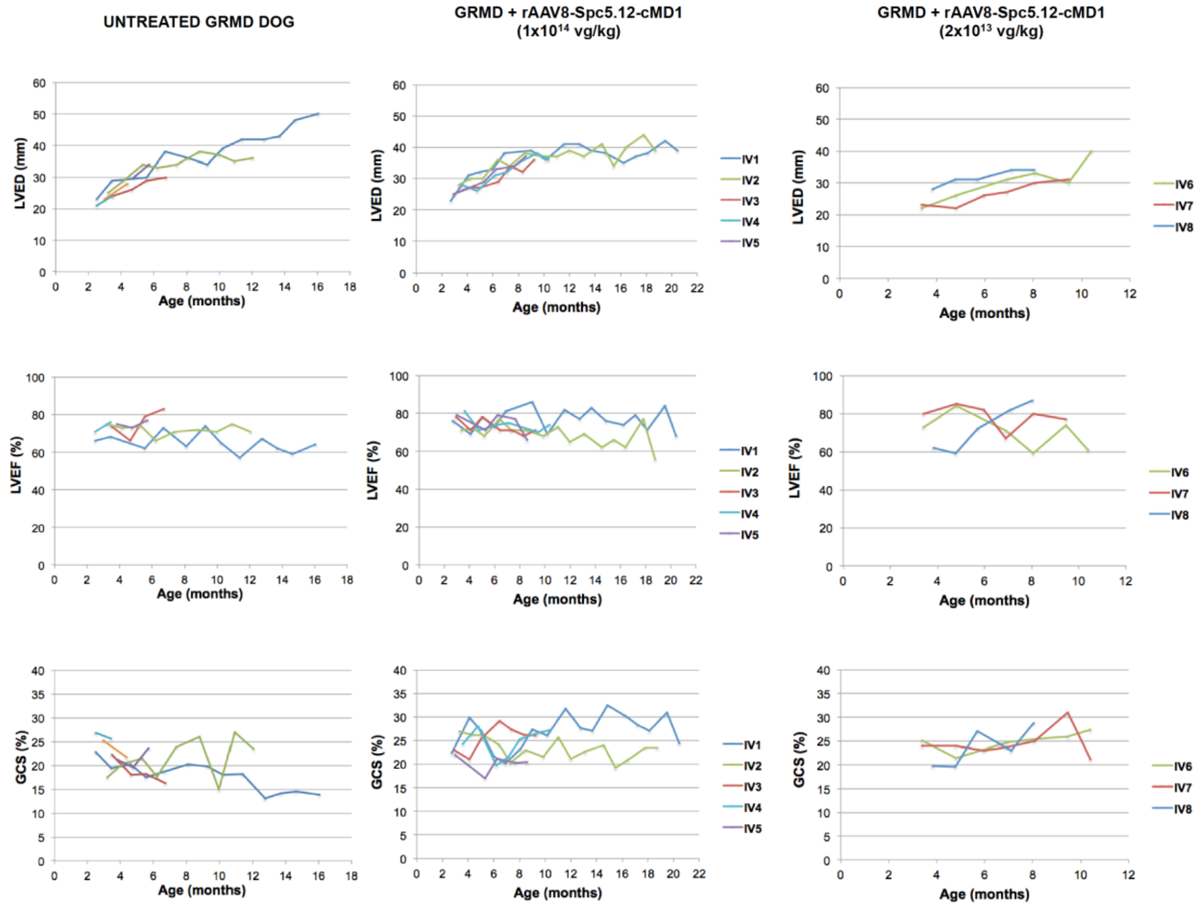

<u>Supplementary Figure 10</u>: Assessment of cardiac function of GRMD dogs injected with rAAV2/8-Spc5.12-cMD1 by the IV route. This analysis was performed using conventional echocardiography, 2D color tissue Doppler imaging and speckle tracking imaging. Results obtained in untreated GRMD dogs (n=6), GRMD dogs injected with $1x10^{14}$ vg/kg (n=5) and GRMD dogs injected with $2x10^{13}$ vg/kg (n=3) for left ventricle end-diastolic diameter (LVED), left ventricle ejection fraction (LVEF) and global circumferential strain at the subendocardial level (GCS) are presented here as representative examples. Since the emergence of cardiac pathology is very rare and with a late onset (over 1 year of age) in the untreated GRMD dogs of our colony, we were not able to show any significant difference between the animals of the different experimental groups.

Figure 1B

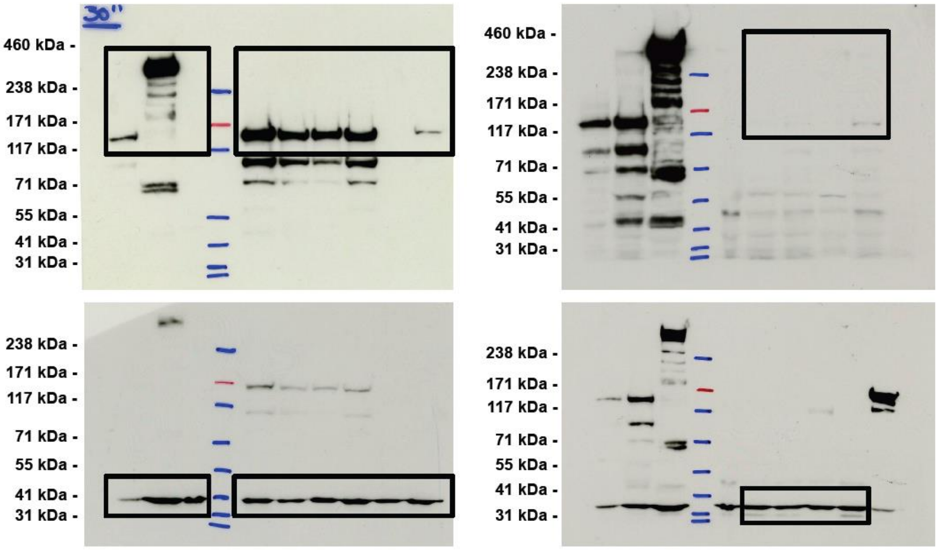

Figure 4D

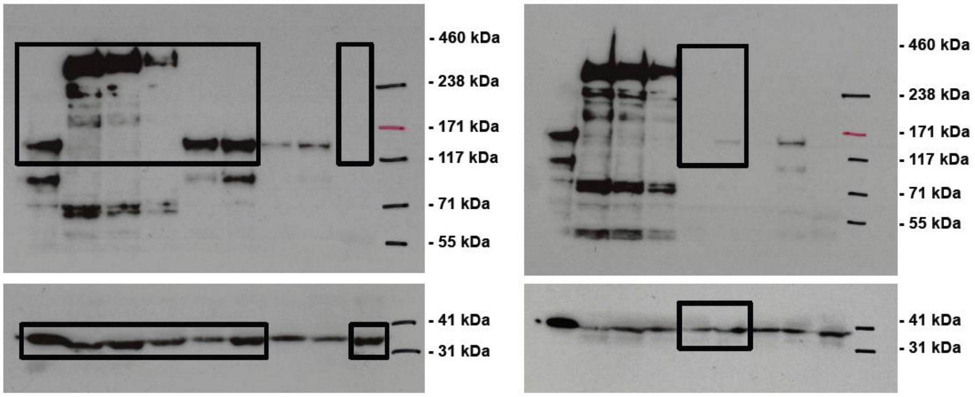

Supplementary figure 8

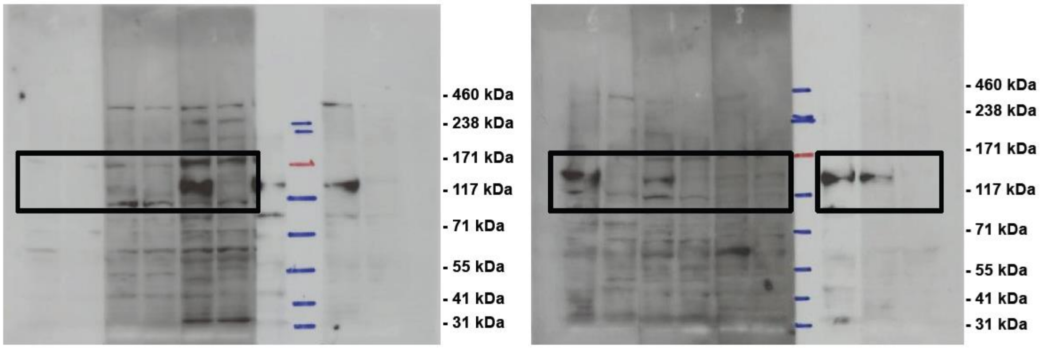

Supplementary Figure 11: Uncropped Western-Blots included in this manuscript.

<u>Supplementary Table 1:</u> Clinical follow up of digestive and respiratory functions in GRMD dogs injected with rAAV2/8-Spc5.12-cMD1 by the IV route.

Group	Dog	Age of dysphagia appearance	Mean age of dysphagia appearance	Age of abdominal breathing appearance	Mean age of abdominal breathing appearance
Group IV-A $1x10^{14}$ vg/kg	IV1	11 months	10 months +/- 2 months	20 months	N/A
	IV2	12 months		None*	
	IV3	7 months		None*	
	IV4	11 months		None*	
	IV5	9 months		None*	
Group IV-B $2x10^{13}$ vg/kg	IV6	4 months	4.6 months +/- 1.2 months	7 months	6.7 months +/- 0.7 month
	IV7	4 months		7 months	
	IV8	6 months		6 months	
Untreated GRMD dogs	Control 1	6 months	5.7 months +/- 0.6 month	5 months	4.7 months +/- 0.6 month
	Control 2	6 months		5 months	
	Control 3	5 months		4 months	

** None at least during the follow-up duration*

(i.e. during 26, 9.5, 10.5 and 10 months after birth respectively for dogs IV2, IV3, IV4 and IV5)

N/A = not applicable

<u>Supplementary Table 2:</u> Anti-AAV8 specific IFN-γ secretion from PBMC of GRMD dogs injected with rAAV2/8-Spc5.12-cMD1 by the LR route

Dog	IFN-γ secretion by PBMC with LV-VP1 AAV8							
	**Bef. inj.**	**Wk +2**	**Wk +3**	**Wk +5**	**Wk +7**	**Wk + 9**	**Wk +13**	**Wk +14/+15**
LR1	nd	nd	/	nd	/	nd	nd	/
LR2	nd	nd	/	nd	/	nd	nd	/
LR3	nd	nd	nd	nd	/	nd	/	nd
LR4	nd	nd	nd	nd	nd	nd	nd	nd

/ = not determined; nd = not detected
